# Characterization of the Largest Effector Gene Cluster of *Ustilago maydis*

**DOI:** 10.1371/journal.ppat.1003866

**Published:** 2014-07-03

**Authors:** Thomas Brefort, Shigeyuki Tanaka, Nina Neidig, Gunther Doehlemann, Volker Vincon, Regine Kahmann

**Affiliations:** Max Planck Institute for Terrestrial Microbiology, Department of Organismic Interactions, Marburg, Germany; Purdue University, United States of America

## Abstract

In the genome of the biotrophic plant pathogen *Ustilago maydis*, many of the genes coding for secreted protein effectors modulating virulence are arranged in gene clusters. The vast majority of these genes encode novel proteins whose expression is coupled to plant colonization. The largest of these gene clusters, cluster 19A, encodes 24 secreted effectors. Deletion of the entire cluster results in severe attenuation of virulence. Here we present the functional analysis of this genomic region. We show that a 19A deletion mutant behaves like an endophyte, i.e. is still able to colonize plants and complete the infection cycle. However, tumors, the most conspicuous symptoms of maize smut disease, are only rarely formed and fungal biomass in infected tissue is significantly reduced. The generation and analysis of strains carrying sub-deletions identified several genes significantly contributing to tumor formation after seedling infection. Another of the effectors could be linked specifically to anthocyanin induction in the infected tissue. As the individual contributions of these genes to tumor formation were small, we studied the response of maize plants to the whole cluster mutant as well as to several individual mutants by array analysis. This revealed distinct plant responses, demonstrating that the respective effectors have discrete plant targets. We propose that the analysis of plant responses to effector mutant strains that lack a strong virulence phenotype may be a general way to visualize differences in effector function.

## Author Summary

In this study, we provide the first step to the functional analysis of the largest gene cluster in the *Ustilago maydis* genome encoding 24 secreted effectors. While the deletion of the entire cluster dramatically affected tumor formation and abolished anthocyanin induction, only one of the genes had a large contribution to tumor formation, while another effector gene was primarily responsible for the anthocyanin induction. Unexpectedly, the cluster mutant could still colonize plants and complete the life cycle, i.e. behaves like an endophyte. Despite only small contributions to tumor formation, individual effector mutants caused distinct plant responses, suggesting that they affect discrete plant processes. On these grounds we are proposing to use plant responses as a general readout to assess and compare effector gene function.

## Introduction

*U. maydis* is a biotrophic fungal pathogen causing smut disease in maize. To cause disease, haploid cells of compatible mating type need to fuse on the plant surface and develop an infectious dikaryon [1], [2]. Upon perception of appropriate surface cues [3], the dikaryon differentiates non-melanized appressoria that penetrate plant cells directly, presumably aided by local secretion of lytic enzymes [4]. During penetration, the host plasma membrane invaginates and encases the fungal hyphae, a feature typical for biotrophs. This establishes an extended interaction interface for the exchange of signals and nutrients [5], [6]. Initial intracellular biotrophic growth of *U. maydis* is followed by intercellular growth during later stages of the infection, concomitant with massive proliferation in mesodermal tissue close to the veins. At this developmental stage, huge fungal aggregates form in cavities between plant cells followed by differentiation of ornamented diploid spores [6]. Fungal proliferation coincides with plant cell enlargement and resumption of mitotic divisions [6]. *U. maydis* can infect and cause symptoms on all above ground maize organs, with the infection staying locally confined. This is in contrast to related smut fungi that show systemic spread throughout the plant but produce symptoms only in the male and female inflorescences [7], [8].

During initial contact of *U. maydis* with the plant leaf and presumably triggered by fungal pathogen-associated molecular patterns (PAMPs), a number of plant defense genes are induced. This upregulation disappears during fungal penetration, suggesting that these initial defense responses are actively suppressed by the fungus during the plant colonization stages [9]. Also, several genes associated with suppression of plant cell death are induced. One of these, the maize cystatin CC9, has recently been functionally analyzed [10]. Silencing of CC9 enhanced maize defense gene expression and upon infection with *U. maydis* a hypersensitive response was observed. CC9 was shown to suppress apoplastic cysteine protease activity, illustrating that CC9 is a novel compatibility factor for the biotrophic interaction of maize with *U. maydis* [10]. After plant colonization the most dramatic transcriptional changes in the host affect hormone signaling, induction of antioxidants, secondary metabolism, as well as a block in the transition from a juvenile sink tissue to a mature, photosynthetically active source tissue normally observed during leaf establishment [9]. The latter supports the observation that *U. maydis* is able to colonize young meristematic maize tissue, but is unable to infect differentiated source tissue [11]. The changes in plant gene expression observed after host colonization are likely to be brought about by secreted fungal effector molecules. The genome of *U. maydis* encodes about 300 novel secreted effectors that are upregulated during plant colonization and largely lack known InterPro domains. Of these a significant percentage is arranged in gene clusters and deletion of entire clusters can have dramatic effects on virulence [12], [13]. Genome comparisons with the related smut fungi *Sporisorium reilianum* and *U. hordei* revealed that the majority of secreted effectors also exist in these relatives. With respect to conservation, effectors fall in two classes: approximately 34% are highly conserved in all smut fungi sequenced so far [8] and the remainder are poorly conserved, reflecting the arms race with the host. Furthermore, in *U. hordei* the tight clustering of effector genes seen in *U. maydis* and *S. reilianum* is largely disrupted [7], [8]. Effector genes in *U. maydis* are plant-induced [12], and work by Skibbe et al. [14] has revealed that the expression of some effector genes is tissue specific, i.e. is different when *U. maydis* colonizes seedlings, adult leaves or tassel, and the need for the corresponding effectors may be restricted to the respective tissues. So far the function of only very few of the many novel effectors has been elucidated in the *U. maydis*/maize pathosystem. Pep1, a conserved effector is needed for penetration [15] and affects plant defense responses by inhibiting apoplastic plant peroxidases [16]. Pit2, another conserved effector affects host defense responses [17] through inhibition of apoplastic plant cysteine proteases [18]. Cmu1 is a secreted chorismate mutase that is taken up by plant cells and lowers salicylic acid (SA) levels in infected tissue through metabolic priming [19].

Here we describe the beginning of the functional analysis of cluster 19A, the largest effector gene cluster in *U. maydis*. In a previous study, the entire cluster 19A comprising 23 putative effector genes in a 40 kb genomic region was deleted [12]. Cluster 19A mutants were severely attenuated in virulence and except for some ligula swelling, the mutants rarely elicited tumor formation [12]. In this study, we map the most important effectors for seedling infection in this cluster, and show that most strains deleted for individual effector genes show only minor reductions in virulence but elicit distinct plant responses.

## Results

### Mapping of cluster 19A genes contributing to tumor formation and anthocyanin induction after seedling infection

**Figure 1:**
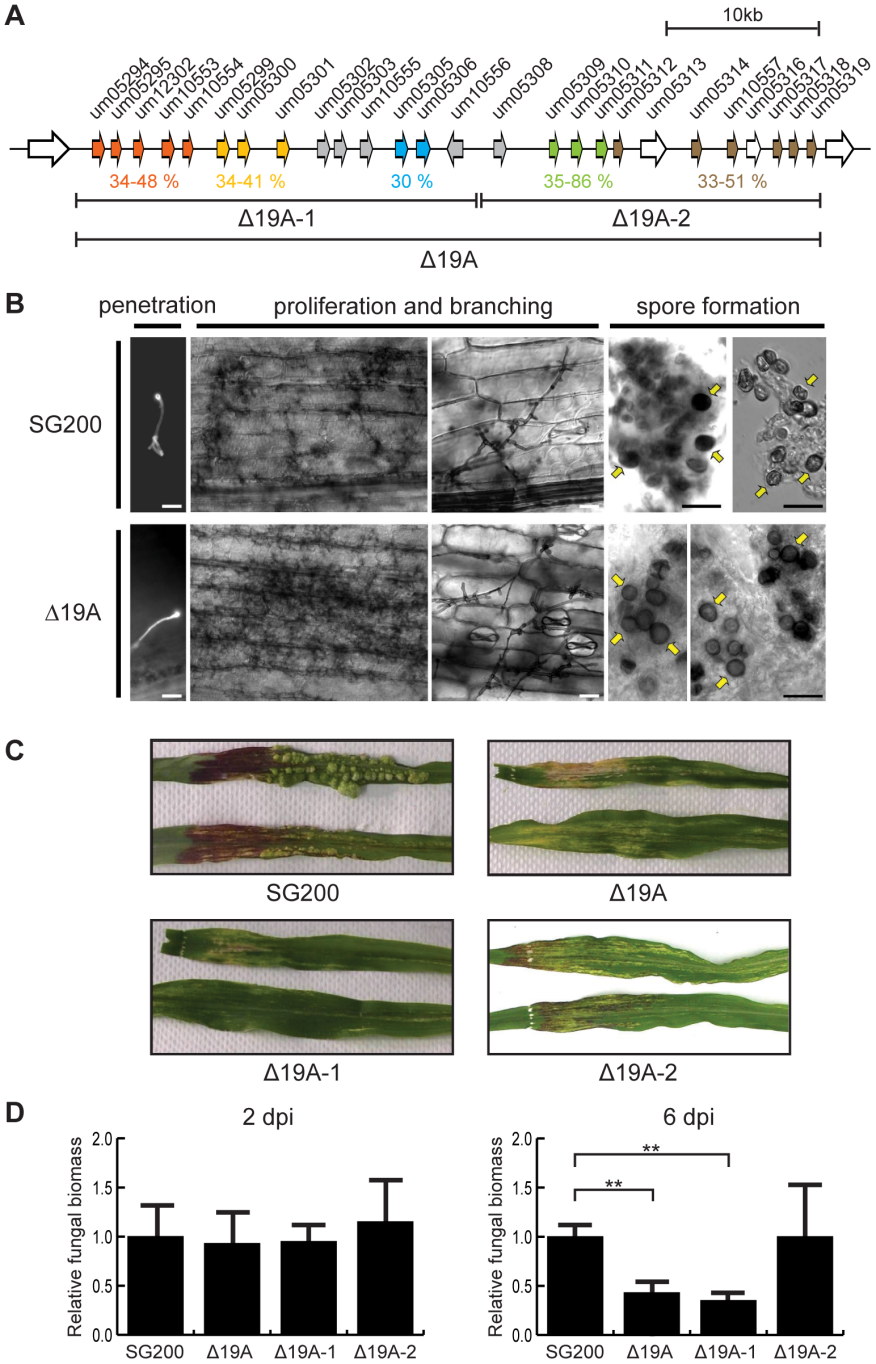
Phenotypic analysis of cluster 19A mutants. A) Schematic representation of the cluster 19A region in the genome of *U. maydis*. Arrows indicate open reading frames (ORFs), labeled by the respective gene numbers. Related gene families are indicated in orange, yellow, blue, green and brown and the percentages of amino acid similarity are given below. Unique effector genes are shown in grey. Genes encoding proteins with a predicted secretion signal are depicted in orange, yellow, blue, green, brown or grey while open arrows indicate genes encoding proteins without a prediction for secretion. The left half of cluster 19A was designated as 19A-1 and the right half was designated 19A-2 as indicated. B) Biotrophic development of SG200 and SG200Δ19A. Maize seedlings were infected with SG200 or SG200Δ19A and development was monitored microscopically. At 1 dpi penetrating hyphae on the leaf surface were visualized by calcofluor staining. Proliferating biotrophic hyphae were observed after chlorazole-black E staining at 13 dpi. At 30 dpi mature teliospores were observed indicated by yellow arrows. Bar = 100 µm. C) Macroscopic symptoms of representative leaves infected with SG200, SG200Δ19A, SG200Δ19A-1 and SG200Δ19A-2 at 12 dpi. Note the absence of anthocyanin pigment accumulation in infections with SG200Δ19A and SG200Δ19A-1. D) Quantification of fungal biomass by qPCR. Genomic DNA was extracted from the leaves infected with SG200, SG200Δ19A, SG200Δ19A-1 and SG200Δ19A-2 at 2 and 6 dpi and used for qPCR. Relative fungal biomass was calculated by the comparison between *U. maydis* peptidylprolyl isomerase gene (*ppi*) and *Z. mays* glyceraldehyde 3-phosphate dehydrogenase gene (*GAPDH*). Error bars indicate standard deviation. ** *p*<0.01. *p*-values were calculated by Student's *t*-test.

Cluster 19A was originally predicted to encode 23 secreted effectors [12]. The manual reannotation based on comparison with *S. reilianum* and *U. hordei* now predicts the presence of 24 effector genes, plus one gene related to a reverse transcriptase (*um05313*) presumably originating from a retrotransposon and one pseudogene (*um05316*) (http://mips.helmholtz-muenchen.de/genre/proj/ustilago) (Figure 1A).

**Figure 2:**
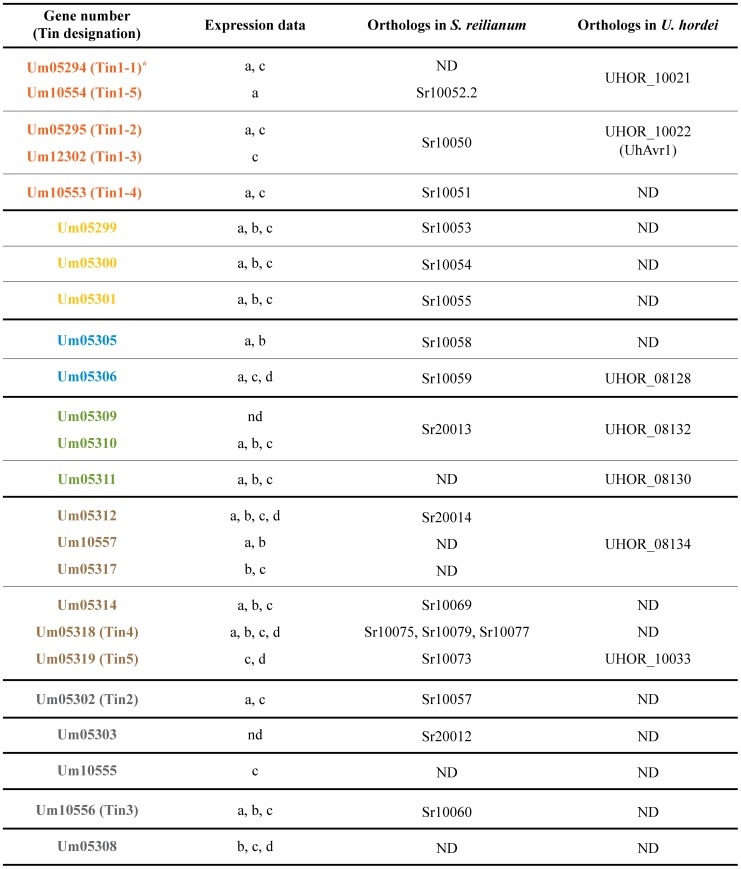
Expression data compilation for cluster 19A genes and presence of orthologous genes in other smut fungi. Expression data for cluster 19A genes were compiled from the following sources: a) Gene expressed in young seedlings [14], b) Gene expressed in tassel [14], c) Gene expressed in tumor tissue [12], d) Gene down-regulated in response to *b* inactivation during biotrophic development [20] and nd) no expression detected. Orthologs in *S. reilianum* and *U. hordei* are listed, and in these columns ND indicates that no orthologs were detected at a cut-off of e-value 10e^−10^. The adopted color scheme corresponds to the scheme used in Figures 1, 3 and 4. Please note that the order of genes was changed in several cases and does not follow the order in cluster 19A (*).

Among the effector genes in cluster 19A, we detect five gene families based on amino acid sequence similarity (Figure 1A, Figure 2 and Figure S1). Um05294, Um05295, Um12302, Um10553 and Um10554 display between 34–48% similarity at the amino acid sequence level (Figure 1A). Another family (Figure 1A) comprises genes *um05299*, *um05300* and *um05301*. The respective proteins show between 34–41% similarity. The two effector proteins encoded by the adjacent genes *um05305* and *um05306* display 30% amino acid similarity (Figure 1A). A three gene family codes for Um05309, Um05310, Um05311 with 35–86% amino acid sequence similarity and the largest family is comprised of Um05312, Um05314, Um10557, Um05317, Um05318 and Um05319 with 33–51% amino acid similarity (Figure 1A). Outside of cluster 19A the *U. maydis* genome does not contain paralogs to any of these gene families. The 24 effectors encoded by cluster 19A do not contain recognizable protein domains nor do they display a characteristic spacing of cysteine residues described for several other *U. maydis* effectors [13]. However, orthologs for most of these genes are found in the genomes of *S. reilianum* and *U. hordei* [7], [8] (http://mips.helmholtz-muenchen.de/genre/proj/sporisorium/) (http://mips.helmholtz-muenchen.de/genre/proj/MUHDB/) (Figure 2). Published expression data for cluster 19A genes from different *U. maydis* infected tissues [12], [14] are compiled in Figure 2. These studies revealed that except for two genes where expression could not be detected, genes in cluster 19A are differentially induced when different plant organs are colonized (Figure 2). Furthermore, only three of the cluster 19A genes are downregulated when the central regulator for pathogenic development, the bE/bW complex, is switched off during biotrophic development [20] (Figure 2). This illustrates, that the individual cluster genes are plant induced but do not appear to be co-regulated.

Given the strong virulence phenotype of cluster 19A mutants [12], we examined whether the cluster 19A mutant is impaired in biotrophic growth. To this end, we compared biotrophic development of the mutant strain SG200Δ19A and the progenitor strain SG200. To our surprise, SG200Δ19A formed appressoria on the plant surface, proliferated inside plant tissue and differentiated teliospores, at a late time point comparable to infections with SG200. This explains, why spores had not been detected in the previous study [12] and illustrated that the 19A mutant could undergo biotrophic development and complete the life cycle (Figure 1B). With respect to macroscopic symptoms, tumor formation was abolished and instead chlorotic and necrotic areas became apparent (Figure 1C). In addition, anthocyanin accumulation was completely abolished in mutant infected tissue, while prominent anthocyanin stained regions were observed in leaves infected by SG200 (Figure 1C).

**Figure 3:**
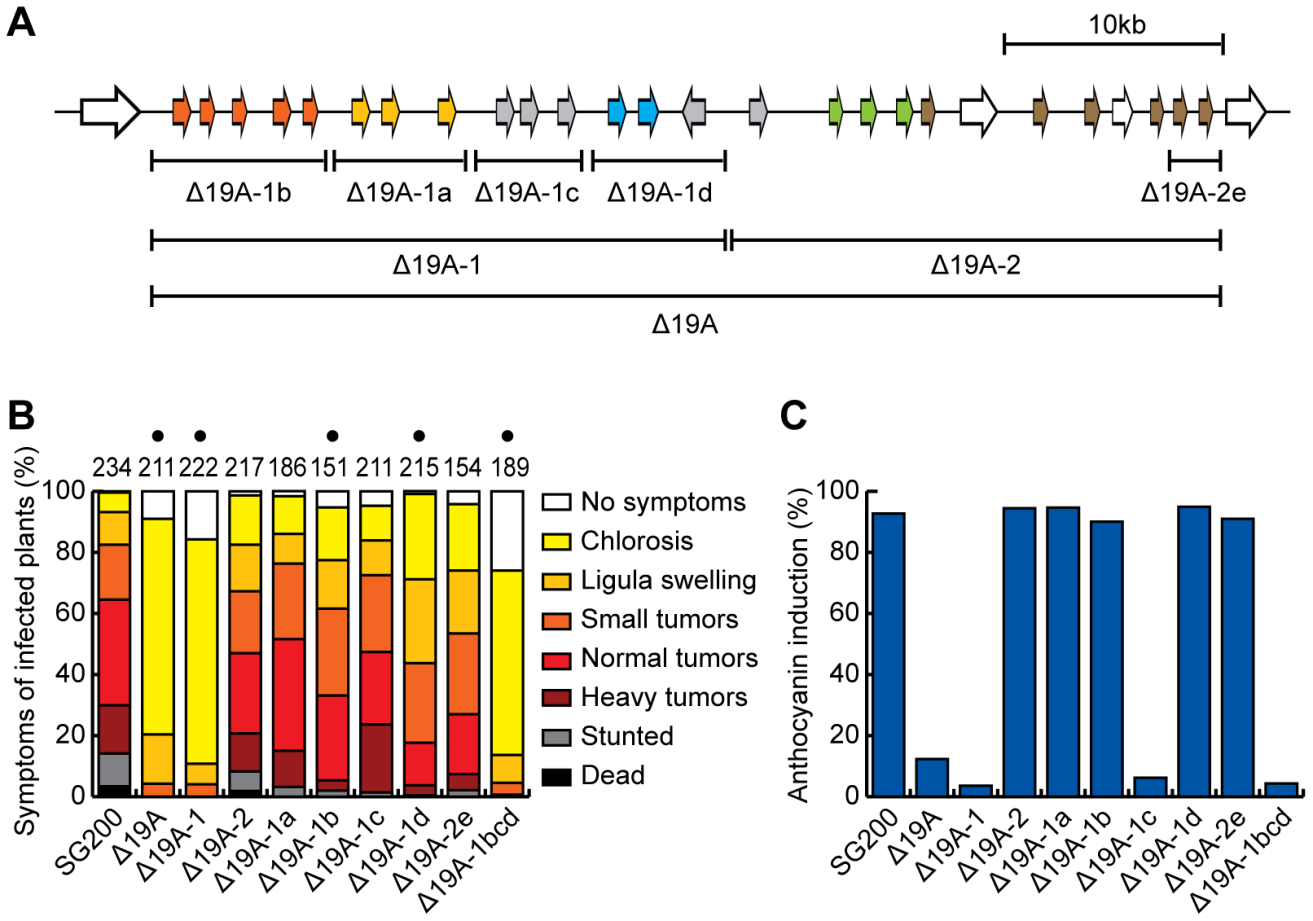
Mapping of effector genes responsible for the virulence phenotype of cluster 19A mutant. A) Schematic representation of analyzed sub-deletions. The region of 19A-1 was divided into 4 parts, 19A-1b, 19A-1a, 19A-1c and 19A-1d according to the distribution of gene families. In the second half of the cluster (19A-2) only the 19A-2e region was deleted. B) Quantitative evaluation of virulence of cluster 19A mutants. The scoring scheme follows the one described by Kaemper et al. [12] with severity of virulence corresponding to the color intensity. The numbers on top of respective bars indicate the total number of infected plants. Results from at least 3 independent experiments are combined. Dots above respective panels indicate that at least one of symptoms (ligula swelling, small tumors, normal tumors, heavy tumors, stunted or dead) was significantly changed in the mutant relative to SG200 by Student's *t*-test. C) Quantification of infected plants showing anthocyanin accumulation. In the same experiments as in B) the percentage of plants showing anthocyanin accumulation was scored.

To identify the genes contributing to the phenotype of the cluster 19A mutant, a series of strains was generated carrying sub-deletions of cluster 19A. At first, we divided cluster 19A into two parts which we designate 19A-1 (left half region of cluster 19A) and 19A-2 (right half region of cluster 19A) (Figure 1A) and generated respective deletion mutants. When tested for phenotype after seedling infection in comparison to SG200, SG200Δ19A-1 showed a dramatic reduction of tumor formation and loss of anthocyanin accumulation. The effects were comparable to infections with SG200Δ19A. Conversely, SG200Δ19A-2 was only weekly attenuated in virulence and was able to elicit anthocyanin accumulation (Figure 1C and Figure 3). To determine possible differences in the efficiency of plant colonization by SG200 and the different derived mutant strains, we analyzed fungal biomass in colonized tissue by quantitative real time PCR using *U. maydis* peptidylprolyl isomerase (*ppi*) and *Z. mays* glyceraldehyde 3-phosphate dehydrogenase (*GAPDH*) as reference genes for quantifying fungal and plant biomass, respectively [15], [21]. At 2 days post infection (dpi), fungal biomass of SG200Δ19A, SG200Δ19A-1 and SG200Δ19A-2 was comparable to the SG200 infection (Figure 1D, left panel). At 6 dpi, however, fungal biomass of SG200Δ19A and SG200Δ19A-1 was lower compared to SG200 (Figure 1D, right panel), suggesting growth defects of these mutant strains at this later time point where massive fungal proliferation inside tumors is observed in SG200 infected tissue. Fungal biomass of SG200Δ19A-2 lacking the right half of cluster 19A was not significantly different from SG200 (Figure 1D), consistent with the weak effect of this deletion on virulence. These results indicated that the major effector genes responsible for the phenotype of the cluster 19A mutant after seedling infection must reside in the left half of cluster 19A.

To determine the contribution to virulence of the 14 genes located in the left half of cluster 19A, we divided the region into four parts, 19A-1a, 19A-1b, 19A-1c and 19A-1d making sure that existing gene families were deleted simultaneously (Figure 3A). Respective deletion mutants were generated and assayed for tumor formation and ability to elicit anthocyanin accumulation (Figure 3B,C). Of these four mutants SG200Δ19A-1a and SG200Δ19A-1c did show a small reduction of tumor formation, but this was not statistically significant (Figure 3B). The deletion of 19A-1b and 19A-1d significantly lowered tumor formation with 19A-1d showing the strongest effect (Figure 3B). Anthocyanin accumulation was abolished by the deletion of 19A-1c and was unaffected in infections with the other sub-deletion mutants (Figure 3C). A triple deletion generated by combining 19A-1b, 19A-1c and 19A-1d deletions resulted in a mutant strain (SG200Δ19A-1bcd) that was severely reduced in tumor formation and failed to induce anthocyanin, comparable to the deletion strain lacking the left half of cluster 19A (SG200Δ19A-1). This firmly establishes that region 19A-1a does not measurably contribute to virulence (Figure 3B).

Based on the finding that the *um05318* gene in *U. maydis* cluster 19A has three paralogous genes at a syntenic position in cluster 19A of *S. reilianum* [7] (Figure 2) that contribute to virulence in *S. reilianum* (H. Ghareeb and J. Schirawski, personal communication), we decided to delete the two rightmost genes in *U. maydis* cluster 19A (19A-2e, Figure 3A). This was done also to rule out that the lack of a virulence phenotype when deleting the entire right half (Δ19A-2, Figure 3B) is caused by balancing positive and negative effects of effector gene deletions on virulence [12]. The resulting strain designated SG200Δ19A-2e showed weakly attenuated tumor formation that was comparable to strain SG200Δ19A-2 deleted for the entire right half of the cluster and this was again not statistically relevant (Figure 3B).

### Identification of major effector genes contributing to cluster 19A deletion phenotypes

**Figure 4:**
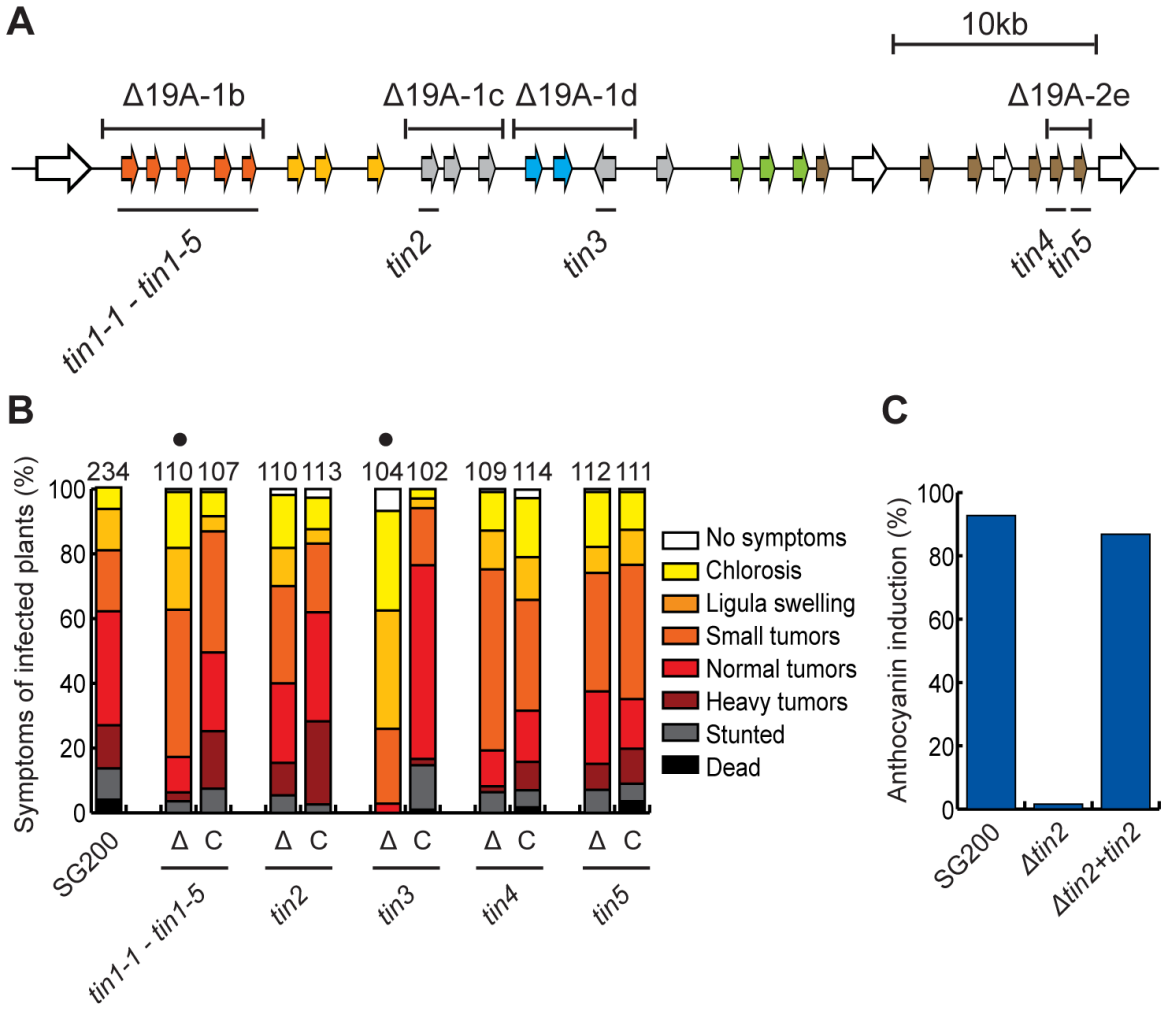
Virulence of *tin* gene deletion mutants and complemented strains. A) Schematic representation of the deleted *tin* genes in cluster 19A. *tin1-1* to *tin1-5* correspond to the 5 genes represented by 19A-1b, the other *tin* genes lie in region 19A-1c (*tin2*), region 19A-1d (*tin3*), and region 19A-2e (*tin4* and *tin5*). B) Quantitative evaluation of virulence of *tin* gene deletion mutants and complemented strains. Virulence of the displayed deletion mutants and respective complementation strains (indicated as Δ and C, respectively below each bar) was determined as described in Figure 3B. C) Quantification of infected plants showing anthocyanin accumulation after infection with *tin2* mutant and control strains. Plants were infected with the indicated strains and the percentage of plants showing anthocyanin accumulation was scored 12 dpi.

To identify the major effector genes that contribute most strongly to the virulence phenotype of sub-clusters 19A-1b, 19A-1c, 19A-1d and 19A-2e, we initially generated overlapping sub-deletions and tested them for virulence (not shown). For example, the cluster 19A-1d region was subdivided into a double deletion of *um05305*/*um05306* and a double deletion of *um05306/um10556*, respectively. These double mutants were tested for virulence and in this case only the double deletion of *um05306/um10556* was affected in virulence, allowing the conclusion that *um10556* is the responsible gene (not shown). This was then followed up by single deletions of the genes identified in this approach (Figure 4A). With respect to 19A-1b comprising five related genes designated *tin1-1 to tin1-5*, we were unable to identify the respective major individual effector gene(s), as the observed effects of further sub-deletions on tumor formation could no longer be assessed as being statistically relevant (data not shown). This suggested that these five members of the same gene family (Figure 2) contribute weakly but additively to virulence. Complementation experiments where all 5 genes were re-introduced in strain SG200Δ19A-1b revealed that the weak virulence phenotype of the 19A-1b deletion could be complemented (Figure 4B). For the region 19A-1c the deletion of *um05302* (designated *tin2*) showed a comparable reduction in virulence to the deletion of the entire 19A-1c region. In addition, the deletion of *tin2* abolished anthocyanin induction (Figure 4B,C). The introduction of a single copy of *tin2* into SG200Δtin2 complemented the weakly reduced tumor formation as well as anthocyanin accumulation (Figure 4B,C). The single deletion of *um10556* (designated *tin3*) significantly affected tumor formation (Figure 4B). Also in this case, tumor formation could be restored to a level comparable to SG200 by introducing *tin3* in single copy (Figure 4B). After infection, the single gene deletion mutant of *um05318* (designated *tin4*) showed a lower incidence of plants with stronger disease symptoms than SG200 infected plants (Figure 4B). Upon complementation, this disease category was increased compared to SG200Δtin4 and more plants showed heavy tumors and were stunted or dead (Figure 4B). This suggests a weak contribution of *tin4* to tumor formation. With respect to *um05319* (designated *tin5*), the single gene deletion had minor effects on virulence and the reintroduction of the gene did not significantly change the disease scores (Figure 4B).

### Plant responses to cluster 19A mutants

The analysis of single effector gene mutants for cluster 19A revealed in general, that deletions of individual genes (with the exception of *tin3*) had only minor or statistically non-substantial effects on virulence (Figure 4B), suggesting that the strong virulence defect observed in the entire cluster 19A deletion is due to additive effects and/or concerted action of several effectors. To visualize this and to obtain evidence whether individual effectors target distinct plant processes, we decided to analyze the plant responses to infection by the 19A deletion strain as well as to several single effector gene mutants on the transcriptome level. Maize seedlings were infected by SG200Δ19A, SG200Δ19A-1b, SG200Δtin3, SG200Δtin4 and SG200Δtin5. RNA was extracted from infected plant material harvested at 4 dpi, a time point where the individual mutants should not differ in fungal biomass as assessed from the analysis of sub-deletions (Figure 1D). Three biological replicates were prepared and analyzed by Affymetrix maize genome microarrays. For technical reasons, the expression data for plants infected with the SG200Δtin2 mutant strain could not be included in this comparative transcriptome analysis.

Maize gene expression profiles of tissue infected with cluster mutant strains were compared to profiles of SG200 infected and mock-treated plants, which had been generated in our previous study on the transcriptional responses of maize to *U. maydis* and had been used as reference in the analysis of plant responses to *pep1* and *pit2* effector mutants [9], [15], [17]. RMA-normalized microarray data were then subjected to a one-way ANOVA and contrast gene lists were generated using a fold change of ±2 and a corrected *p*-value of 0.05 as cutoffs (Table S1). Expression of a set of 13 genes differentially regulated after infection with different mutant strains was subsequently analyzed by quantitative real-time PCR (qRT-PCR) with RNA from independently generated infected plant material, and this allowed validating the array results (Figure S2).

**Figure 5:**
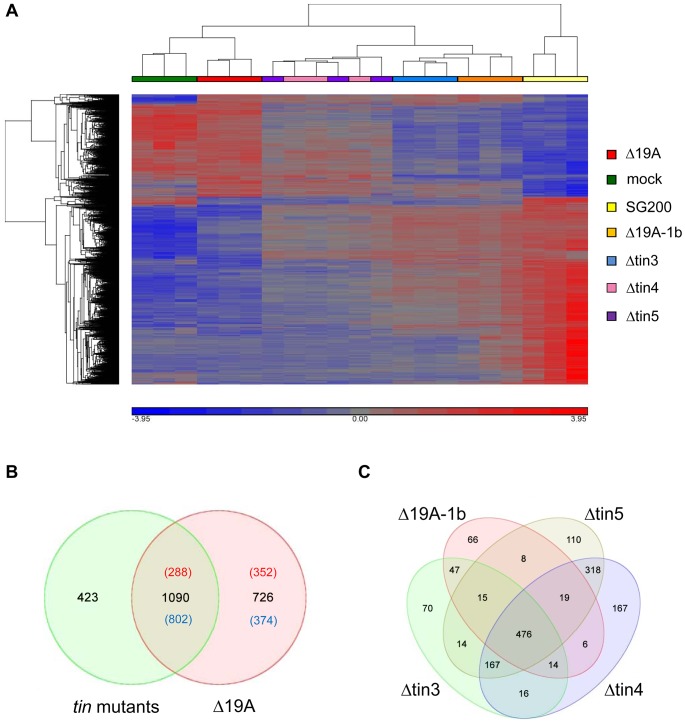
Plant responses to cluster 19A mutant strains. A) Hierarchical clustering visualizing the relative expression of the 1816 maize genes transcriptionally regulated 4 days after infection by *U. maydis* strain SG200Δ19A. X-axis depicts clustering of the microarray samples, with three biological replicates per strain. Y-axis shows clustering of the regulated maize transcripts based on the similarity of their expression patterns. B) Venn diagram showing numbers of maize transcripts differentially expressed 4 days after infection with SG200Δ19A (red circle) as compared to the combined deregulated transcripts of all individual *tin* mutants (green circle). For the *tin* mutants, all genes regulated in at least one of the mutant infections have been combined. Red numbers indicate induced expression; numbers of down-regulated transcripts are given in blue. C) Venn diagram showing maize transcripts differentially expressed 4 days after infection by the *U. maydis* mutants SG200Δ19A-1b (Δ19A-1b), SG200Δtin3 (Δtin3), SG200Δtin4 (Δtin4), SG200Δtin5 (Δtin5), respectively.

Compared to SG200 infected samples, 1816 maize genes were differentially regulated in response to SG200Δ19A (Table S1). A hierarchical clustering of these 1816 genes was performed for the whole data set to visualize the relations between the transcriptional responses to the individual *U. maydis* strains (Figure 5A). As expected, the maximal distance in gene expression was between SG200 infections and infections by the SG200Δ19A mutant, which caused only very weak disease symptoms and thus displayed the highest similarity to the mock-inoculated plants (Figure 5A). On the other hand SG200Δ19A-1b infections showed highest similarity to SG200 infections, illustrating that the 5 *tin1* effector genes have only a weak contribution to plant responses, which is in line with their weak effect on virulence. Profiles of plant responses to strains carrying *tin* gene deletions clearly discriminated SG200Δtin3 and SG200Δ19A-1b, while responses to SG200Δtin4 and SG200Δtin5 infections were not separated by the hierarchical clustering (Figure 5A), indicating that similar responses were elicited by *tin4* and *tin5* mutants.

We also combined the transcriptional responses elicited by each of the four individual *tin* mutant strains to be able to compare this to the response elicited by SG200Δ19A, the strain carrying the full deletion of cluster 19A and to reveal contributions of genes not deleted individually. In total, 1513 maize genes were differentially regulated by the four *tin* mutants compared to SG200 infections, while 1816 genes were differentially regulated after infection with the cluster deletion mutant SG200Δ19A (Figure 5B). Interestingly, a comparison of these combined “responses to individual *tin* gene deletions” to the SG200Δ19A responsive genes showed only a partial overlap of differentially regulated transcripts (Figure 5B). Of the 726 genes differentially expressed in response to the whole cluster mutant but not detected in the “responses to individual *tin* gene deletions” 352 genes were induced and 374 genes were repressed (Figure 5B). Among the induced transcripts, particularly biotin synthesis genes were induced specifically in SG200Δ19A infected tissue, while plant cellulose synthesis genes were downregulated after infection by the Δ19A deletion strain (Table S2 and Figures S3 and S4). In addition, several anthocyanin biosynthesis related genes were downregulated after infection with the cluster 19A mutant while they were not included as differentially expressed in “responses to individual *tin* gene deletions” (Table S2). This most likely indicates a contribution of the Tin2 effector to anthocyanin induction, and reflects that plant responses to the *tin2* deletion strain could not be included in “responses to individual *tin* gene deletions” for technical reasons. Of the 1090 “shared differentially regulated plant genes”, 288 genes were induced, while 802 genes were downregulated (Figure 5B). Induced genes comprised pathogen response genes such as *PR4*, *PR5* and several oxidases, demonstrating an elevated plant defense in response to *tin* gene and cluster 19A mutant strains (Table S2). Downregulated transcripts were strongly enriched for genes involved in DNA-metabolism and DNA-modification, particularly histones and DNA-methyltransferases (Table S2 and Figure S5). This most likely reflects the reduced tumor formation observed in all the mutants compared to SG200. On the other hand, 423 genes were differentially regulated after infections with the *tin* mutant strains (Figure 5B) but these were not differentially regulated after infection with the cluster 19A mutant. Among these 423 genes, several chitinases and peroxidases were found (Table S2).

To get clues on the possible roles of the *tin* genes during host colonization we next visualized the differentially regulated plant genes in response to individual *tin* mutants. This analysis revealed that 476 maize genes were commonly regulated by all four mutants, while 1027 genes only responded to a subset of *tin* mutants (Figure 5C). The smallest number of specifically regulated maize genes was found after infections with the SG200Δ19A-1b mutant, which lacks the five related *tin1* effector genes, i.e. this mutant shared 73% (476 out of 651) of differentially regulated genes with all other strains. Amongst the 66 plant genes that were specifically regulated after SG200Δ19A-1b infections, four maize endochitinase genes were significantly induced compared to SG200 infected leaves. In addition, transcript levels of two salicylic acid binding proteins and peroxidase-12, which was found to be involved in the maize apoplastic oxidative burst [16], were induced (Table S3). This suggests that *tin1* genes modulate basal defenses.

The *tin3* mutant specifically affected the differential regulation of 70 maize genes (Table S3). Conspicuously, sucrose synthase and several transcription factors including auxin-response factors were induced suggesting a link to the reduced ability for tumor formation.

After infections by *tin4* and *tin5* mutant strains the majority of differentially expressed maize genes were shared (Figure 5C), which is in accordance with the hierarchical clustering result which places these two strains closely together (Figure 5A). Nevertheless, 167 genes were only differentially regulated by the *tin4* mutant and 110 maize transcripts were differentially regulated in infections by the *tin5* mutant (Figure 5C and Table S3). Although *tin4* and *tin5* mutants displayed an indistinguishable virulence behavior, they elicited distinct molecular responses in maize. This appears to be a general feature and for example also holds for a strain carrying the 19A-1b deletion (Figure 3B) where virulence is only weakly attenuated. When the 10 most strongly induced host genes are compared after SG200Δ19A-1b infection, only one gene was among the top 10 genes upregulated after infection with SG200Δtin3, SG200Δtin4 or SG200Δtin5, respectively (Table S1).

**Figure 6:**
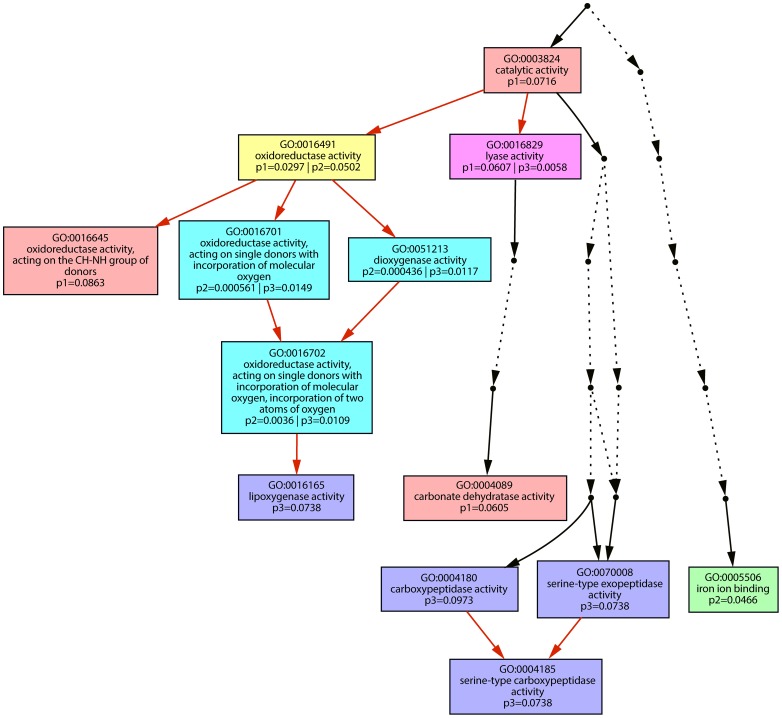
Graphic representation of Gene Ontology terms. Hierarchical presentation of Gene Ontology [43] terms showing molecular functions significantly enriched amongst upregulated maize transcripts in *tin* mutant infected tissue. Numbers give *p*-values for enrichment of the respective GO. p1: *p*-value of enrichment in upregulated genes after infection by SG200Δtin3 (pink boxes); p2: *p*-value of enrichment in upregulated maize genes after infection by SG200Δtin4 (green box); p3: *p*-value of enrichment in upregulated maize genes after infection by SG200Δtin5 (blue boxes). Box in yellow indicates significant enrichment in samples p1 and p2; box in dark pink indicates significant enrichment in p1 and p3; boxes in turquoise indicate significant enrichment in p2 and p3 and darker color symbolizes more significant enrichment. Red arrows indicate hierarchical connections between (significantly enriched) Gene Ontology terms. Dotted arrows represent GO terms not enriched and therefore spared out from the figure.

Gene ontology enrichment analyses were performed for the plant genes upregulated after infection by the three single *tin* gene deletion strains SG200Δtin3, SG200Δtin4 and SG200Δtin5 (Figures S6, S7, S8). Differentially enriched functions for the individual effector deletion strains were visualized and in addition genes corresponding to the enriched functions are listed (Figure 6 and Table S4). This revealed that statistically distinct processes were induced in plants infected by the different mutants. For SG200Δtin3, oxidoreductases and carbonate dehydratases were enriched, while SG200Δtin4 infection induced plant genes significantly enriched for functions involved in iron ion binding. In SG200Δtin5 infection, on the other hand, lipoxygenases and serine-carboxypeptidases were induced, which were not as highly upregulated in the other mutants (Figure 6 and Table S4). These results illustrate that plant responses to effector mutants which show no or only weak reductions in macroscopic symptoms can be highly specific and can be used to describe and discriminate mutant phenotypes.

## Discussion

In this communication, we have dissected the largest *U. maydis* effector gene cluster 19A, identified the most relevant effectors for seedling infection and present evidence, that individual effectors target distinct processes in the host plant. It was published before, that the deletion of cluster 19A abolishes tumor formation [12]. We now show that this dramatic phenotype is not associated with a block in biotrophic development. The cluster 19A mutant was still able to complete the life cycle up to the formation of teliospores. However, massive fungal proliferation observed at later stages in tumor tissue in infections with wild type strains was absent, suggesting that the effectors in this cluster are responsible for tumor induction either directly or indirectly but not for growth per se in the infected tissue. The analysis of plant responses elicited by the whole cluster mutant (SG200Δ19A) in comparison to responses to the progenitor strain SG200 revealed that 1816 of the 13,339 maize genes represented on the chip were differentially regulated. The analysis of the maize transcriptome changes observed for the individual effector mutant infections revealed that about 60% of these changes were shared by all mutants, suggesting that they are unspecific. Another aspect of the comparative transcriptome profiling was the finding that there is incomplete overlap between the genes affected by the individual mutants and genes altered in their regulation when the entire cluster is deleted. This could indicate that the effects of individual effector deletions cease to be visible when the entire cluster is deleted, i.e. the dramatic phenotype of the cluster deletion might bury the more subtle physiological changes caused by individual mutants. Our study has also revealed that effectors not studied individually because of their undetectable contribution to virulence after seedling infection, may profoundly affect the metabolic activity of the infected tissue. An example is the observed upregulation of biotin biosynthesis after infection with SG200Δ19A which is not observed in any of the *tin* mutant infections. In *Arabidopsis thaliana* it has been shown that biotin is critical for suppressing spontaneous cell death [22]. The fact that biotin biosynthesis appears upregulated may indicate a direct involvement of a specific effector for maintaining a certain level of this essential cofactor. Alternatively, biotin upregulation could be a secondary effect that allows the cluster 19A mutant to grow in the infected tissue. Future array analyses with mutants of cluster 19A not studied here with respect to the plant responses they elicit should allow to separate primary and secondary effects and allow to uncover the effector responsible for the regulation of glycolysis and biotin biosynthesis, respectively.

Among the common genes differentially expressed after infection with all *tin* mutant strains, we observe an enrichment of upregulated plant defense genes and downregulation of genes involved in DNA metabolism. This is likely to reflect insufficient suppression of plant defenses due to reduced fungal proliferation (or the absence of certain effectors) and reduced plant tumor formation, respectively. Similarly, genes for photosynthesis components were consistently higher expressed after infections with all mutant strains compared to SG200 infections. This is unlikely to reflect an induction of photosynthesis during mutant infections but presumably results from an incomplete shutdown of photosynthesis, usually observed during *U. maydis* wild type infection [9]. The reduction in plant cell wall biosynthesis gene expression after infection with the cluster 19A mutant, which is not seen in infections with individual *tin* mutants, likely reflects the reduced ability of the cluster mutant to induce tumors containing enlarged plant cells [15], while individual *tin* mutants can still induce tumors.

With a minor impact on tumor formation, Tin2 was specifically responsible for anthocyanin accumulation in infected tissue while all other individual mutants showed anthocyanin induction. The transcriptome analysis revealed that several anthocyanin biosynthesis genes were upregulated in all the individual *tin* mutant strains but not after infection with SG200Δ19A lacking the whole cluster including *tin2* (Table S1). This suggests that Tin2 is directly responsible for inducing these anthocyanin biosynthesis genes. Anthocyanin has been hypothesized to have a protective role against abiotic stresses [23], and can be induced after biotic stress, although its role here is unclear [24].

While the virulence assays for *tin4* and *tin5* mutant strains were largely uninformative because of limited assay sensitivity, the transcriptome analysis revealed that *tin4* and *tin5* mutants elicited a series of plant responses that were mutant specific, but in addition 318 differentially regulated plant genes were differentially regulated by *tin4* as well as *tin5* mutant strains. Based on the fact that Tin4 and Tin5 share 19% identity and 39% amino acid similarity this could indicate that these effectors are in the process of diversification to different functions (167 *tin4* specifically regulated transcripts, 110 *tin5* specifically regulated transcripts) while still maintaining some of the original common functions (318 commonly regulated transcripts). This interpretation would also make sense in view of the fact that *um05312*, *um05314*, *um10557* and *um05317* which are also related to *tin4* and *tin5* (Fig. S1E) were not individually deleted in this study. If all of the genes in this family had redundant functions, we would not have expected to see differences in the host responses to the *tin4* and *tin5* mutants. The gene ontology enrichment analysis showed that several genes involved in iron metabolism/uptake were specifically upregulated when *tin4* is missing. Elevated iron availability may directly affect the activity of the respiratory burst oxidase requiring a heme prosthetic group to generate superoxide [25]. Such effects on plant defense have also been described after infections by *Erwinia crysanthemi* [26] and *Blumeria graminis* f. sp. *tritici* [27]. After *tin5* mutant infections lipoxygenases and serine-carboxypeptidases were specifically upregulated, and both types of enzymes have been associated with defense [28], [29]. OsBISCPL1, a serine-carboxypeptidase from rice, was up-regulated in incompatible interactions between rice and the blast fungus, and was implicated in regulation of defense responses from heterologous expression studies [30]. In *S. reilianum* three orthologs of *tin4* are present (*sr10075*, *sr10077* and *sr10079*; Figure 2). The simultaneous deletion of the neighboring genes *sr10073*, *sr10075*, *sr10077* and *sr10079* weakly affected *S. reilianum* virulence (H. Ghareeb and J. Schirawski, personal communication), similar to what we observe for the *tin4, tin5* double mutant of *U. maydis*. As in *U. maydis*, the left half of cluster 19A contributes most strongly to virulence in *S. reilianum* (H. Ghareeb and J. Schirawski, personal communication). Based on the observation that cluster 19A effector genes of *U. maydis* are not essential for tumor formation in tassel (although tumor size in tassel was reduced after infection with SG200Δ19A) [14], we consider organ-specificity of effector function more likely to explain this finding. As *S. reilianum* does not induce tumors in leaves and develops disease symptoms only in the cob and in the tassel, this species may not need effector genes like *tin3* for tumor induction in vegetative tissues of the maize plant. Consistent with this is our observation that the *U. maydis* cluster 19A mutant lacking all 24 effectors can still show biotrophic growth in plant tissue and thus behaves like *S. reilianum* (except for the systemic spread). Interestingly, two of the Tin4 orthologs of *S. reilianum*, *sr10075* and *sr10077*, were recently shown to suppress apical dominance after maize infection (H. Ghareeb, F. Drechsler, C. Löfke, T. Teichmann and J. Schirawski, personal communication). The effect on apical dominance is a late phenotype observed about six weeks after infection of maize plants with *S. reilianum*, i.e. a time point not covered by our assays. To ascertain whether Tin4, Sr10075 and Sr10077 have conserved functions it would be interesting to test whether *tin4* of *U. maydis* can complement the apical dominance phenotype of the respective *S. reilianum* mutants.

Tin1-1 to Tin1-5 is a group of weakly related *U. maydis* effectors, which could not be functionally separated because their individual effects on virulence were too small to be reliably detected. The transcriptome changes of plants infected with a mutant lacking all five related genes revealed specific, strong inductions of endochitinases, SA-binding proteins and the apoplastic peroxidase POX12. POX12 was recently shown to be inhibited by the *U. maydis* effector Pep1 leading to a suppression of the PAMP-triggered oxidative burst [16]. In addition, an NBS-LRR class disease resistance gene (Zm.3568.1) that could be involved in PAMP perception, showed transcriptional induction specifically after SG200Δ19A-1b infections. Together, these changes indicate an enhanced defense response against the 19A-1b deletion mutant, which suggests that the Tin1-1 to Tin1-5 effectors contribute to the suppression of basal host immunity. Interestingly, the immune response triggering avirulence factor, UhAvr1 (UHOR_10022, Figure 2), of *U. hordei* is most closely related to the *U. maydis* effector Tin1-2 and Tin1-3 [31]. With respect to virulence no specific contribution of UhAvr1 could be detected [31], which may be consistent with the very small contribution to virulence that is seen when all five *tin1* genes are deleted.

Tin3 is the effector in cluster 19A, which contributes most strongly to virulence. The strong transcriptional induction of two sucrose synthases after infection with the *tin3* mutant strain (as well as after infection with the cluster 19A mutant) is likely to reflect enhanced photosynthetic activity in contrast to infections with SG200 where the transition from a juvenile sink tissue to a mature, photosynthetically active source tissue is blocked in infected leaves [9]. If an interplay between sucrose and auxin signaling, which was established in *A. thaliana* [32] also exists in maize; this could explain the observed upregulation of several auxin response factors after infection with these mutant strains. The specific upregulation of a WRKY transcription factor after infection with the cluster 19A mutant as well as the *tin3* mutant could indicate elevated defense responses [33], which are downregulated by Tin3 after infections with wild type strains. Alternatively, this regulatory gene might negatively control cell cycle and/or cell expansion, a feature of *U. maydis* induced tumors [9]. Another gene exclusively upregulated after infection with strains deleted for *tin3* or cluster 19A is cytokinin oxidase 3, an enzyme involved in cytokinin degradation. Cytokinin oxidases have been shown to restrict cell division and to regulate the sink capacity of kernels [34]. Thus, the downregulation of these activities by Tin3, presumably after uptake of Tin3 by plant cells, might be necessary for tumor development.

The finding of discrete plant responses after infection with individual effector mutants provides important leads for the functional analyses that can now be followed. For example, the predicted changes in hormone levels attributed to Tin3 could be determined from metabolic profiles or directly connected with Tin3 by transiently expressing Tin3 in plants with appropriate reporter gene constructs. The expression of Tin3 in transgenic plants might even allow assessing, whether the predicted effects on photosynthesis are direct or indirect.

In more general terms our analyses reveal the power of studying pathogen effector mutants, by combining virulence assays with an assessment of plant responses to these mutants. Such comparisons do not only reveal common plant responses that reflect central processes targeted by the infection but in addition provide specific leads to the function of individual effectors. Furthermore, this approach does not rely on a significant virulence phenotype of the effector mutants studied and may thus be highly useful for the analysis of the vast majority of eukaryotic pathogen effectors that fall into this class [35], [36].

## Materials and Methods

### Fungal strains, growth conditions and plant infections

*U. maydis* strains were grown in YEPSL (0.4% yeast extract, 0.4% peptone, 2% sucrose) with shaking at 28°C at 200 rounds min^−1^ (rpm), to an optical density (OD_600_) of 0.6–0.8. Cells were centrifuged at 3000 *g* for 5 min, resuspended in H_2_O to an OD_600_ of 1 and used for syringe infection of 7-day-old maize seedlings (variety Early Golden Bantam, Olds Seeds, Madison). At least 3 biological replicates were tested for virulence and disease was scored 12 dpi following described protocols [12]. To demonstrate the statistical differences of disease symptoms in the mutants compared to SG200 each of the symptoms was tested by Student's *t*-test (** *p*-values<0.01.) and corrected by Bonferroni correction for multiple testing. The haploid solopathogenic strain SG200 [12] was used for virulence assays and all mutations were introduced into this background.

### Strain construction

Standard molecular cloning strategies and techniques were applied [37]. All *U. maydis* strains (Table S5) are derived from the solopathogenic strain SG200 and were generated by a PCR-based gene replacement approach using primers listed in Table S6 or, for complementation experiments, by insertion of p123 derivatives into the *ip* locus as described [38]. Deletion endpoints are depicted in the respective Figures. Constructs used for complementation contained the respective gene plus the promoter region extending up to the next gene plus the Tnos terminator. All generated constructs were sequenced prior to *U. maydis* transformation (Table S7). Isolated *U. maydis* transformants were tested for integration events in the desired loci by southern analysis. For the complementation constructs, single copy integrations into the *ip* locus were selected by southern analysis. For 3 strains only derivatives containing two inserts could be obtained, this is marked in the strain list (Table S5).

### Quantitative real time PCR

For quantification of relative fungal biomass in infected maize leaves 7-day-old maize seedlings were infected with SG200, SG200Δ19A, SG200Δ19A-1 and SG200Δ19A-2 and a section of the third leaf between 1 and 3 cm below the injection site was harvested after 2 dpi and 6 dpi. For genomic DNA extraction leaf material was frozen in liquid nitrogen, ground to powder, and extracted using a phenol-based protocol modified from Hoffman and Winston [39]. The qRT-PCR analysis was performed using an iCycler (Bio-Rad) in combination with the Platinum SYBR Green Supermix (Invitrogen). *U. maydis* biomass was quantified with primers PPI-fw (5′-ACATCGTCAAGGCTATCG-3′) and PPI-re (5′-AAAGAACACCGGACTTGG-3′) amplifying the fungal *ppi* gene. Maize glyceraldehyde dehydrogenase was amplified with primers GAPDH-F (5′- CTTCGGCATTGTTGAGGGTTTG-3′) and GAPDH-R (5′- TCCTTGGCTGAGGGTCCGTC-3′) [15] and served as reference gene for normalization. Relative amounts of fungal DNA (ppi) were then calculated relative to the amount of *GAPDH* DNA using the cycle threshold (Ct)2^−ΔΔCt^ method [40]. Three biological replicates were combined and *p*-values were determined by using Student's *t*-test (** *p*-values<0.01.).

To validate the expression data of the microarray experiment 13 maize genes differentially regulated after infection with different mutant strains were subsequently analyzed by qRT-PCR. Infected plant material was generated as described for the microarray experiment and used for RNA extraction with Trizol (Invitrogen, Karlsruhe, Germany). After extraction, the first-strand cDNA synthesis kit (Invitrogen) was used to reverse transcribe 3 µg of total RNA with oligo(dT) Primers. The qRT-PCR analysis was performed using an iCycler (Bio-Rad) in combination with the SYBR Green Supermix (Invitrogen). Primers used for quantification of maize gene transcription levels are listed in Table S6. Gene expression levels were calculated relative to *GAPDH* expression levels using the cycle threshold (Ct)2^−ΔΔCt^ method [40].

### DNA microarray analysis

For the microarray experiments, maize plants (Early Golden Bantam) grown under defined conditions in a growth chamber were infected with SG200Δ19A-1b, SG200Δtin2, SG200Δtin3, SG200Δtin4 and SG200Δtin5 as described previously [15]. Samples of infected tissue were collected 4 dpi by excising a section of the third leaf between 1 and 3 cm below the injection site. For RNA extraction, material from >20 plants per experiment was combined, ground to powder on constant liquid nitrogen and RNA was extracted with Trizol (Invitrogen, Karlsruhe, Germany). RNA was purified applying the RNeasy kit (Qiagen, Hilden, Germany). Affymetrix maize genome microarrays were performed in three biological replicates, using standard Affymetrix protocols (Midi_Euk2V3 protocol on GeneChip Fluidics Station 450; scanning on Affymetrix GSC3000G). Expression data were submitted to GeneExpressionOmnibus (http://www.ncbi.nlm.nih.gov/geo/) (Accession Number: GSE48406).

Previously published Affymetrix data for SG200 infections [9] (GEO accession Number: GSE10023) and the microarrays performed in this study were analyzed together using the Partek microarray software suite version 6.12. Expression values were normalized using the RMA method. Criteria for significance were a corrected *p*-value (per sample) with a FDR of 0.05 and a fold-change of >2. Differentially expressed genes were calculated by a 1-way ANOVA model using method of moments [41].

### Microscopy

For microscopic analysis of different life cycle stages of *U. maydis* strains SG200 and SG200Δ19A, a section of the third leaf between 1 and 3 cm below the injection site was excised after 1 dpi, 13 dpi and 30 dpi. We used a Zeiss Axiophot with differential interference contrast (DIC) optics for microscopic observations. The pictures were taken using a CCD camera (C4742-95, Hamamatsu). To visualize penetration events, appressoria were stained with calcofluor white (100 µg/ml; Fluorescent Brightener 28, Sigma-Aldrich, Deisenhofen) for 1 min. Intracellular growing fungal hyphae were stained with chlorazol black E using an established protocol [42].

## Supporting Information

Figure S1 Amino acid sequence alignment of gene families on cluster 19A. Amino acid sequences of gene families located on cluster 19A were aligned by Clustal Omega program. Similar amino acid residue among all was highlighted as black box. Similar amino acid residue among several amino acid sequences was highlighted by grey. (A) Orange group, (B) Yellow group, (C) Blue group, (D) Green group, (E) Brown group, described in Figure 1A.
(PPTX)

Figure S2 Quantitative real-time PCR of the genes upregulated in the leaves infected with *tin* mutants. The genes upregulated in the leaves infected with *tin* mutants were picked up from microarray data. RNA samples were extracted from the leaves inoculated with H_2_O or infected with SG200 and *tin* mutants, which were prepared independently, at 4 dpi, and used for qRT-PCR. Error bars were calculated from three biological replicates.
(PPTX)

Figure S3 Gene ontology enrichment analysis of maize genes induced by the cluster 19A deletion but not by the *tin* mutants at 4 dpi. The GOEAST software toolkit [43] was used to identify GO terms for cellular processes (yellow boxes) that are specifically enriched in maize leaves infected with *U. maydis* strain SG200Δ19A samples. Darker color shades indicate higher significance of enrichment. *p*-values are indicated in brackets.
(PPTX)

Figure S4 Gene ontology enrichment analysis of maize genes downregulated by the cluster 19A deletion but not by the *tin* mutants at 4 dpi. The GOEAST software toolkit [43] was used to identify GO terms for cellular processes (yellow boxes) that are enriched in maize leaves infected with *U. maydis* strain SG200Δ19A. Darker color shades indicate higher significance of enrichment. *p*-values are indicated in brackets.
(PPTX)

Figure S5 Gene ontology enrichment analysis of maize genes downregulated by the *tin* mutants and the cluster 19A deletion at 4 dpi. The GOEAST software toolkit [43] was used to identify GO terms for cellular processes that are enriched in maize leaves infected with *U. maydis* strain SG200Δ19A and the *tin* mutants compared to mock-treated samples. Yellow boxes indicate processes enriched in both SG200Δ19A and the *tin* mutants infected samples. Darker color shades indicate higher significance of enrichment. *p*-values are indicated in brackets.
(PPTX)

Figure S6 Gene ontology enrichment analysis of maize genes induced after infection with *U. maydis* strain SG200Δtin3 at 4 dpi. The GOEAST software toolkit [43] was used to identify GO terms for cellular processes (yellow boxes) that are specifically enriched in maize leaves infected with *U. maydis* strain SG200Δtin3 Darker color shades indicate higher significance of enrichment. *p*-values are indicated in brackets.
(PPTX)

Figure S7 Gene ontology enrichment analysis of maize genes induced after infection with *U. maydis* strain SG200Δtin4 at 4 dpi. The GOEAST software toolkit [43] was used to identify GO terms for cellular processes (yellow boxes) that are specifically enriched in maize leaves infected with *U. maydis* strain SG200Δtin4. Darker color shades indicate higher significance of enrichment. *p*-values are indicated in brackets.
(PPTX)

Figure S8 Gene ontology enrichment analysis of maize genes induced after infection with *U. maydis* strain SG200Δtin5 at 4 dpi. The GOEAST software toolkit [43] was used to identify GO terms for cellular processes (yellow boxes) that are specifically enriched in maize leaves infected with *U. maydis* strain SG200Δtin5. Darker color shades indicate higher significance of enrichment. *p*-values are indicated in brackets.
(PPTX)

Table S1 Differentially regulated maize genes after infection with SG200, SG200Δ19A and SG200-derived *tin* mutant strains.
(XLSX)

Table S2 Differentially expressed maize genes shared and not shared in response to the cluster 19A deletion mutant and different *tin* gene deletion mutants.
(XLSX)

Table S3 Differentially regulated maize genes specific to infection with individual *tin* mutant strains.
(XLSX)

Table S4 List of Gene Ontology terms and enriched transcripts.
(XLSX)

Table S5 *U. maydis* strains used in this study.
(DOCX)

Table S6 PCR primers used in this study.
(DOCX)

Table S7 Plasmids used in this study.
(DOCX)

## References

[1] Brefort T, Doehlemann G, Mendoza-Mendoza A, Reissmann S, Djamei A, et al (2009) *Ustilago maydis* as a pathogen. Annu Rev Phytopathol 47: 423–445. 19400641 10.1146/annurev-phyto-080508-081923

[2] Vollmeister E, Schipper K, Baumann S, Haag C, Pohlmann T, et al (2012) Fungal development of the plant pathogen *Ustilago maydis* . FEMS Microbiol Rev 36: 59–77. 21729109 10.1111/j.1574-6976.2011.00296.x

[3] Mendoza-Mendoza A, Berndt P, Djamei A, Weise C, Linne U, et al (2009) Physical-chemical plant-derived signals induce differentiation in *Ustilago maydis* . Mol Microbiol 71: 895–911. 19170880 10.1111/j.1365-2958.2008.06567.x

[4] Schirawski J, Bohnert HU, Steinberg G, Snetselaar K, Adamikowa L, et al (2005) Endoplasmic reticulum glucosidase II is required for pathogenicity of Ustilago maydis. Plant Cell 17: 3532–3543. 16272431 10.1105/tpc.105.036285 PMC1315386

[5] Bauer R, Oberwinkler F, Vanky K (1997) Ultrastructural markers and systematics in smut fungi and allied taxa. Can J Bot 75: 1273–1314.

[6] Doehlemann G, Wahl R, Vranes M, de Vries RP, Kaemper J, et al (2008) Establishment of compatibility in the *Ustilago maydis*/maize pathosystem. J Plant Physiol 165: 29–40. 17905472 10.1016/j.jplph.2007.05.016

[7] Schirawski J, Mannhaupt G, Muench K, Brefort T, Schipper K, et al (2010) Pathogenicity determinants in smut fungi revealed by genome comparison. Science 330: 1546–1548. 21148393 10.1126/science.1195330

[8] Laurie JD, Ali S, Linning R, Mannhaupt G, Wong P, et al (2012) Genome comparison of barley and maize smut fungi reveals targeted loss of RNA silencing components and species-specific presence of transposable elements. Plant Cell 24: 1733–1745. 22623492 10.1105/tpc.112.097261 PMC3442566

[9] Doehlemann G, Wahl R, Horst RJ, Voll LM, Usadel B, et al (2008) Reprogramming a maize plant: transcriptional and metabolic changes induced by the fungal biotroph *Ustilago maydis* . Plant J 56: 181–195. 18564380 10.1111/j.1365-313X.2008.03590.x

[10] van der Linde K, Hemetsberger C, Kastner C, Kaschani F, van der Hoorn RAL, et al (2012) A maize cystatin suppresses host immunity by inhibiting apoplastic cysteine proteases. Plant Cell 24: 1285–1300. 22454455 10.1105/tpc.111.093732 PMC3336116

[11] Wenzler H, Meins F (1987) Persistent changes in the proliferative capacity of maize leaf tissues induced by *Ustilago* infection. Physiol Mol Plant Pathol 30: 309–319.

[12] Kaemper J, Kahmann R, Boelker M, Ma L-J, Brefort T, et al (2006) Insights from the genome of the biotrophic fungal plant pathogen *Ustilago maydis* . Nature 444: 97–101. 17080091 10.1038/nature05248

[13] Mueller O, Kahmann R, Aguilar G, Trejo-Aguilar B, Wu A, et al (2008) The secretome of the maize pathogen *Ustilago maydis* . Fungal Genet Biol 45: S63–S70. 18456523 10.1016/j.fgb.2008.03.012

[14] Skibbe DS, Doehlemann G, Fernandes J, Walbot V (2010) Maize tumors caused by *Ustilago maydis* require organ-specific genes in host and pathogen. Science 328: 89–92. 20360107 10.1126/science.1185775

[15] Doehlemann G, van der Linde K, Assmann D, Schwammbach D, Hof A, et al (2009) Pep1, a secreted effector protein of *Ustilago maydis*, is required for successful invasion of plant cells. PLoS pathogens 5: e1000290–e1000290. 19197359 10.1371/journal.ppat.1000290 PMC2631132

[16] Hemetsberger C, Herrberger C, Zechmann B, Hillmer M, Doehlemann G (2012) The *Ustilago maydis* effector Pep1 suppresses plant immunity by inhibition of host peroxidase activity. PLoS Pathog 8: e1002684 10.1371/journal.ppat.1002684 22589719 PMC3349748

[17] Doehlemann G, Reissmann S, Assmann D, Fleckenstein M, Kahmann R (2011) Two linked genes encoding a secreted effector and a membrane protein are essential for Ustilago maydis-induced tumour formation. Mol Microbiol 81: 751–766. 21692877 10.1111/j.1365-2958.2011.07728.x

[18] Mueller AN, Ziemann S, Treitschke S, Assmann D, Doehlemann G (2013) Compatibility in the *Ustilago maydis*-maize interaction requires inhibition of host cysteine proteases by the fungal effector Pit2. PLoS Pathog 9: e1003177 10.1371/journal.ppat.1003177 23459172 PMC3573112

[19] Djamei A, Schipper K, Rabe F, Ghosh A, Vincon V, et al (2011) Metabolic priming by a secreted fungal effector. Nature 478: 395–398. 21976020 10.1038/nature10454

[20] Wahl R, Zahiri A, Kaemper J (2010) The *Ustilago maydis b* mating type locus controls hyphal proliferation and expression of secreted virulence factors *in planta* . Mol Microbiol 75: 208–220. 19943901 10.1111/j.1365-2958.2009.06984.x

[21] van der Linde K, Kastner C, Kumlehn J, Kahmann R, Doehlemann G (2011) Systemic virus-induced gene silencing allows functional characterization of maize genes during biotrophic interaction with *Ustilago maydis* . New Phytol 189: 471–483. 21039559 10.1111/j.1469-8137.2010.03474.x

[22] Li J, Brader G, Helenius E, Kariola T, Palva ET (2012) Biotin deficiency causes spontaneous cell death and activation of defense signaling. Plant J 70: 315–326. 22126457 10.1111/j.1365-313X.2011.04871.x

[23] Chalker-Scott L (1999) Environmental significance of anthocyanins in plant stress responses. Photochem Photobiol 70: 1–9.

[24] Steyn WJ, Wand SJE, Holcroft DM, Jacobs G (2002) Anthocyanins in vegetative tissues: a proposed unified function in photoprotection. New Phytol 155: 349–361. 10.1046/j.1469-8137.2002.00482.x 33873306

[25] Vignais PV (2002) The superoxide-generating NADPH oxidase: structural aspects and activation mechanism. Cell Mol Life Sci 59: 1428–1459. 12440767 10.1007/s00018-002-8520-9 PMC11337443

[26] Segond D, Dellagi A, Lanquar V, Rigault M, Patrit O, et al (2009) NRAMP genes function in *Arabidopsis thaliana* resistance to *Erwinia chrysanthemi* infection. Plant J 58: 195–207. 19121106 10.1111/j.1365-313X.2008.03775.x

[27] Liu G, Greenshields DL, Sammynaiken R, Hirji RN, Selvaraj G, et al (2007) Targeted alterations in iron homeostasis underlie plant defense responses. J Cell Sci 120: 596–605. 17244651 10.1242/jcs.001362

[28] Joo Y-C, Oh D-K (2012) Lipoxygenases: Potential starting biocatalysts for the synthesis of signaling compounds. Biotechnol Adv 30: 1524–1532. 22537875 10.1016/j.biotechadv.2012.04.004

[29] Feng Y, Xue Q (2006) The serine carboxypeptidase like gene family of rice (*Oryza sativa* L. ssp *japonica*). Funct Integr Genomic 6: 14–24. 10.1007/s10142-005-0131-8 15809843

[30] Liu H, Wang X, Zhang H, Yang Y, Ge X, et al (2008) A rice serine carboxypeptidase-like gene OsBISCPL1 is involved in regulation of defense responses against biotic and oxidative stress. Gene 420: 57–65. 18571878 10.1016/j.gene.2008.05.006

[31] Ali S, Laurie JD, Linning R, Cervantes-Chávez JA, Gaudet D, et al (2014) An immunity-triggering effector from the barley smut fungus *Ustilago hordei* resides in an Ustilaginaceae-specific cluster bearing signs of transposable element-assisted evolution. PLoS Pathog 10: e1004223. 24992661 10.1371/journal.ppat.1004223 PMC4081816

[32] Stokes ME, Chattopadhyay A, Wilkins O, Nambara E, Campbell MM (2013) Interplay between sucrose and folate modulates auxin signaling in arabidopsis. Plant Physiol 162: 1552–1565. 23690535 10.1104/pp.113.215095 PMC3707552

[33] Miller G, Shulaev V, Mittler R (2008) Reactive oxygen signaling and abiotic stress. Physiol Plantarum 133: 481–489. 10.1111/j.1399-3054.2008.01090.x 18346071

[34] Massonneau A, Houba-Hérin N, Pethe C, Madzak C, Falque M, et al (2004) Maize cytokinin oxidase genes: differential expression and cloning of two new cDNAs. J Exp Bot 55: 2549–2557. 15475375 10.1093/jxb/erh274

[35] Rafiqi M, Ellis JG, Ludowici VA, Hardham AR, Dodds PN (2012) Challenges and progress towards understanding the role of effectors in plant-fungal interactions. Curr Opin Plant Biol 15: 477–482. 22658704 10.1016/j.pbi.2012.05.003

[36] Win J, Krasileva KV, Kamoun S, Shirasu K, Staskawicz BJ, et al (2012) Sequence divergent RXLR effectors share a structural fold conserved across plant pathogenic oomycete species. PLoS Pathog 8: e1002400 10.1371/journal.ppat.1002400 22253591 PMC3257287

[37] Sambrook J, Fritsch EF, Maniatis T (1989) Molecular Cloning: A Laboratory Manual. Cold Spring Harbor, NY: Cold Spring Harbor Laboratory Press.

[38] Loubradou G, Brachmann A, Feldbrugge M, Kahmann R (2001) A homologue of the transcriptional repressor Ssn6p antagonizes cAMP signalling in *Ustilago maydis* . Mol Microbiol 40: 719–730. 11359577 10.1046/j.1365-2958.2001.02424.x

[39] Hoffman CS, Winston F (1987) A ten-minute DNA preparation from yeast efficiently releases autonomous plasmids for transformation of *E. coli* . Gene 57: 267–272. 3319781 10.1016/0378-1119(87)90131-4

[40] Livak KJ, Schmittgen TD (2001) Analysis of relative gene expression data using real-time quantitative PCR and the 2^((−ΔΔC(T))^ Method. Methods 25: 402–408. 11846609 10.1006/meth.2001.1262

[41] Eisenhart C (1947) The assumptions underlying the analysis of variance. Biometrics 3: 1–21. 20240414

[42] Brachmann A, Schirawski J, Muller P, Kahmann R (2003) An unusual MAP kinase is required for efficient penetration of the plant surface by *Ustilago maydis* . EMBO J 22: 2199–2210. 12727886 10.1093/emboj/cdg198 PMC156070

[43] Zheng Q, Wang X-J (2008) GOEAST: a web-based software toolkit for Gene Ontology enrichment analysis. Nucleic Acids Res 36: W358–W363. 18487275 10.1093/nar/gkn276 PMC2447756

