# Supplementary material for: Characterization of the Largest Effector Gene Cluster of Ustilago maydis
Source: PLoS Pathog. 2014 Jul 3;10(7):e1003866. doi: 10.1371/journal.ppat.1003866 (PMC4081774; doi:10.1371/journal.ppat.1003866)
Supplement: Figure S1 — Amino acid sequence alignment of gene families on cluster 19A. Amino acid sequences of gene families located on cluster 19A were aligned by Clustal Omega program. Similar amino acid residue among all was highlighted as black box. Similar amino acid residue among several amino acid sequences was highlighted by grey. (A) Orange group, (B) Yellow group, (C) Blue group, (D) Green group, (E) Brown group, described in Figure 1A. (PPTX) [file ppat.1003866.s001.pptx]

## Slide 1
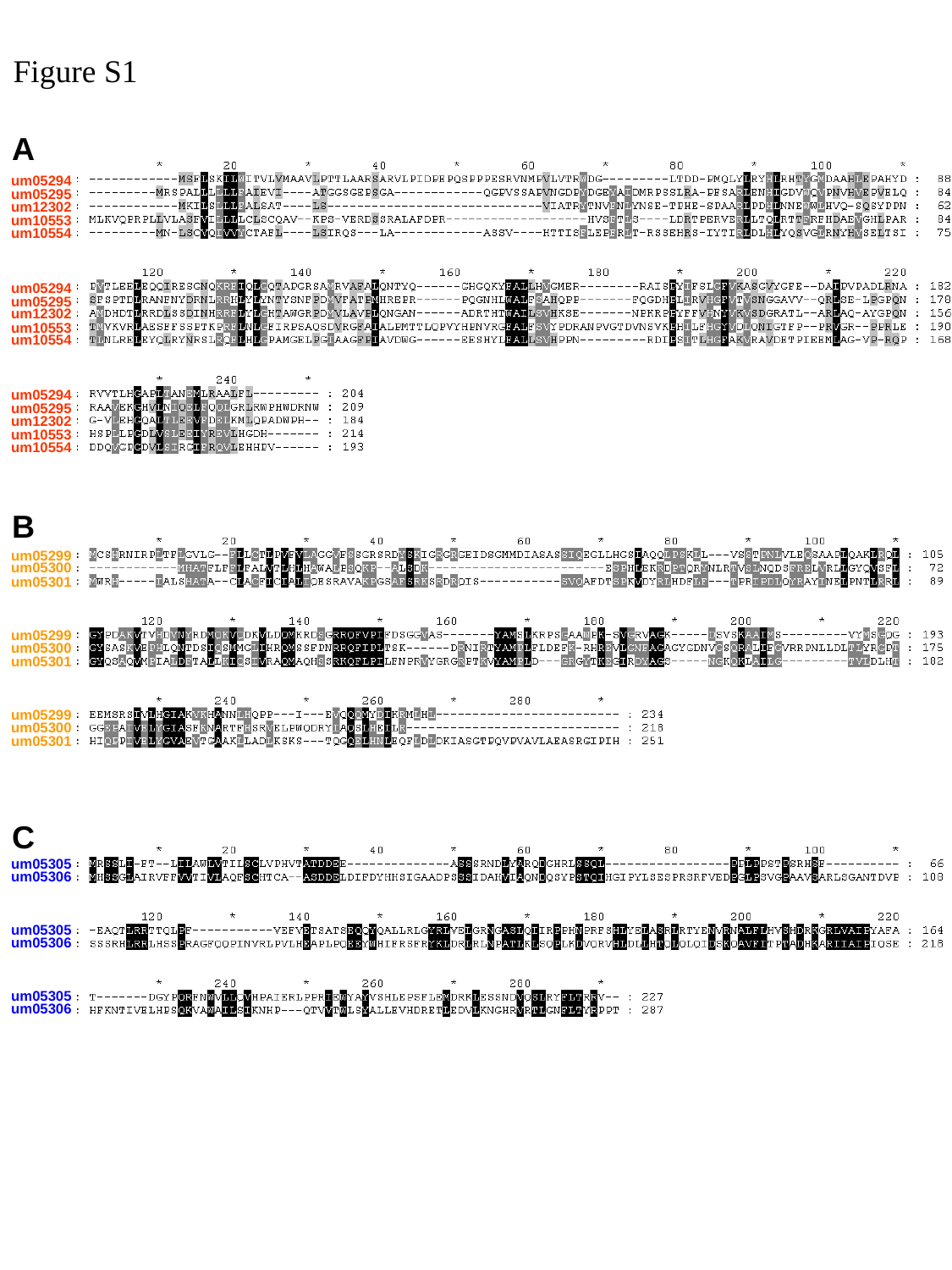

Figure S1
A
um05294
um05295
um12302
um10553
um10554
um05294
um05295
um12302
um10553
um10554
um05294
um05295
um12302
um10553
um10554
B
um05299
um05300
um05301
um05299
um05300
um05301
um05299
um05300
um05301
C
um05305
um05306
um05305
um05306
um05305
um05306

## Slide 2
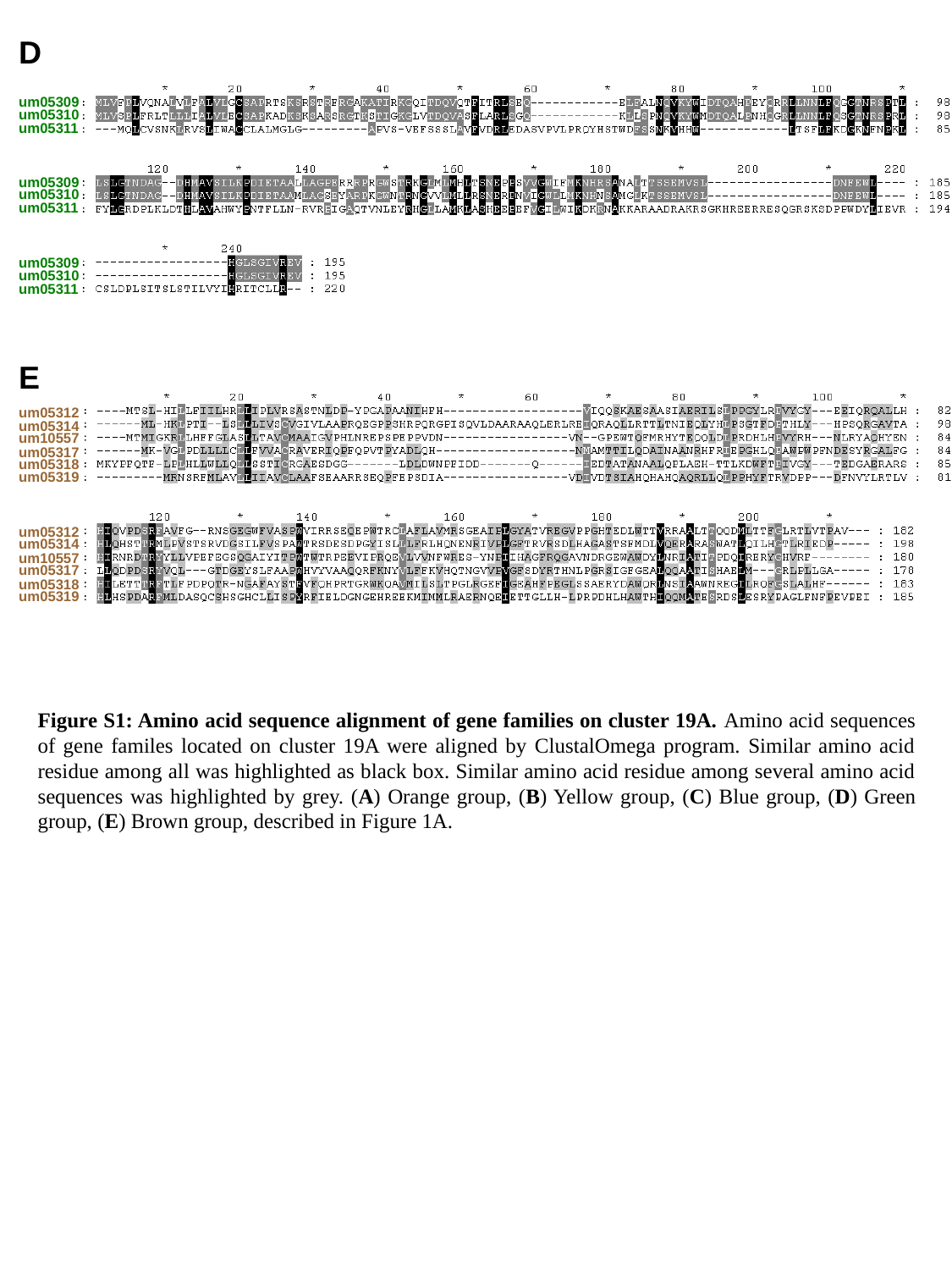

D
um05309
um05310
um05311
um05309
um05310
um05311
um05309
um05310
um05311
E
um05312
um05314
um10557
um05317
um05318
um05319
um05312
um05314
um10557
um05317
um05318
um05319
Figure S1: Amino acid sequence alignment of gene families on cluster 19A. Amino acid sequences of gene familes located on cluster 19A were aligned by ClustalOmega program. Similar amino acid residue among all was highlighted as black box. Similar amino acid residue among several amino acid sequences was highlighted by grey. (A) Orange group, (B) Yellow group, (C) Blue group, (D) Green group, (E) Brown group, described in Figure 1A.
