# Supplementary material for: Characterization of the Largest Effector Gene Cluster of Ustilago maydis
Source: PLoS Pathog. 2014 Jul 3;10(7):e1003866. doi: 10.1371/journal.ppat.1003866 (PMC4081774; doi:10.1371/journal.ppat.1003866)
Supplement: Figure S2 — Quantitative real-time PCR of the genes upregulated in the leaves infected with tin mutants. The genes upregulated in the leaves infected with tin mutants were picked up from microarray data. RNA samples were extracted from the leaves inoculated with H2O or infected with SG200 and tin mutants, which were prepared independently, at 4 dpi, and used for qRT-PCR. Error bars were calculated from three biological replicates. (PPTX) [file ppat.1003866.s002.pptx]

## Slide 1
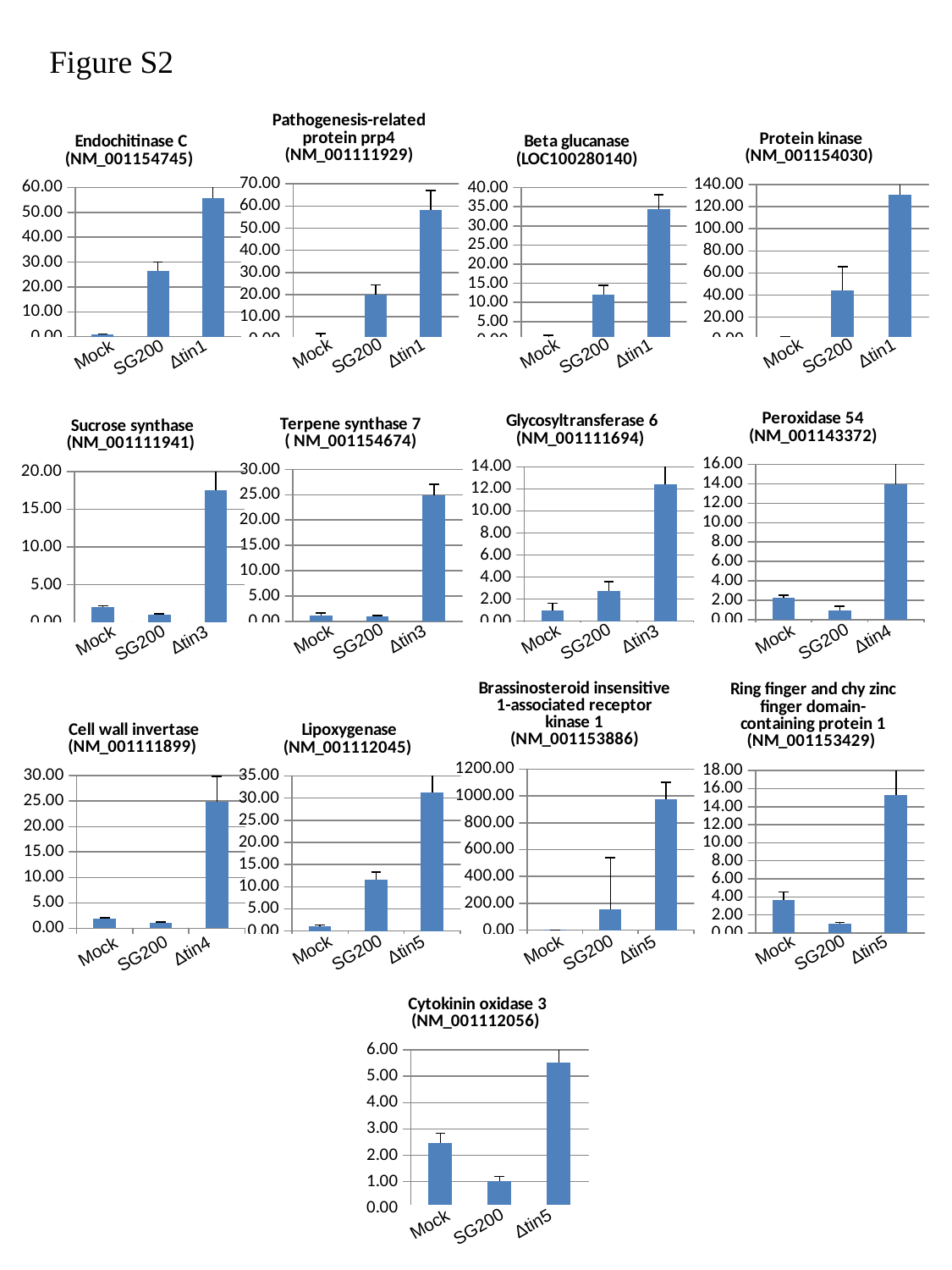

Figure S2
### Chart: Pathogenesis-related protein prp4
(NM_001111929)
| Category | prp4 (C) |
|---|---|
| MOCK | 1.0 |
| SG200 | 20.15873679831783 |
| tin1 | 58.350239267725755 |
### Chart: Protein kinase
(NM_001154030)
| Category | protein kinase (H) |
|---|---|
| MOCK | 1.0 |
| SG200 | 44.22121215896856 |
| tin1 | 130.99185817558669 |
### Chart: Beta glucanase
(LOC100280140)
| Category | beta glucanase (D) |
|---|---|
| MOCK | 1.0 |
| SG200 | 12.125732532083182 |
| tin1 | 34.29675080116157 |
### Chart: Endochitinase C
(NM_001154745)
| Category | endochitinase C (B) |
|---|---|
| MOCK | 1.0 |
| SG200 | 26.599612676569077 |
| tin1 | 55.71523605095191 |Δtin1
Δtin1
Δtin1
Δtin1
Mock
Mock
Mock
Mock
SG200
SG200
SG200
SG200
### Chart: Peroxidase 54
(NM_001143372)
| Category | pox54 (D) |
|---|---|
| MOCK | 2.2449240966187354 |
| SG200 | 1.0 |
| tin4 | 13.928809012737924 |
### Chart: Glycosyltransferase 6
(NM_001111694)
| Category | Glycosiltransferase 6 (C) |
|---|---|
| MOCK | 1.0 |
| SG200 | 2.763825759935576 |
| tin3 | 12.409158094669873 |
### Chart: Terpene synthase 7
( NM_001154674)
| Category | Terpene synthase (C) |
|---|---|
| MOCK | 1.2030250360821098 |
| SG200 | 1.0 |
| tin3 | 24.81831618933973 |
### Chart: Sucrose synthase
(NM_001111941)
| Category | Suc synthase (2nd run) (B) |
|---|---|
| MOCK | 2.0 |
| SG200 | 1.0 |
| tin3 | 17.549199675113968 |Δtin3
Δtin4
Δtin3
Δtin3
Mock
Mock
Mock
Mock
SG200
SG200
SG200
SG200
### Chart: Brassinosteroid insensitive 1-associated receptor kinase 1
(NM_001153886)
| Category | BRI1-associated receptor kinase 1(G) |
|---|---|
| MOCK | 1.0 |
| SG200 | 153.98720461851084 |
| tin5 | 977.7578024042664 |
### Chart: Ring finger and chy zinc finger domain-containing protein 1
(NM_001153429)
| Category | Ring finger protein (G) |
|---|---|
| MOCK | 3.6468899542328597 |
| SG200 | 1.0 |
| tin5 | 15.277465662566629 |
### Chart: Lipoxygenase
(NM_001112045)
| Category | Lipoxygenase (2nd run) (F) |
|---|---|
| MOCK | 1.0 |
| SG200 | 11.57815389952307 |
| tin5 | 31.26911898989591 |
### Chart: Cell wall invertase
(NM_001111899)
| Category | cell wall invertase (E) |
|---|---|
| MOCK | 1.8234449771164298 |
| SG200 | 1.0 |
| tin4 | 24.81831618933979 |Δtin5
Δtin5
Δtin5
Δtin4
Mock
Mock
Mock
Mock
SG200
SG200
SG200
SG200
### Chart: Cytokinin oxidase 3
(NM_001112056)
| Category | Cytokinin oxidase 3 (H) |
|---|---|
| MOCK | 2.4622888266898166 |
| SG200 | 1.0 |
| tin5 | 5.5276515198710845 |Δtin5
Mock
SG200

## Slide 2
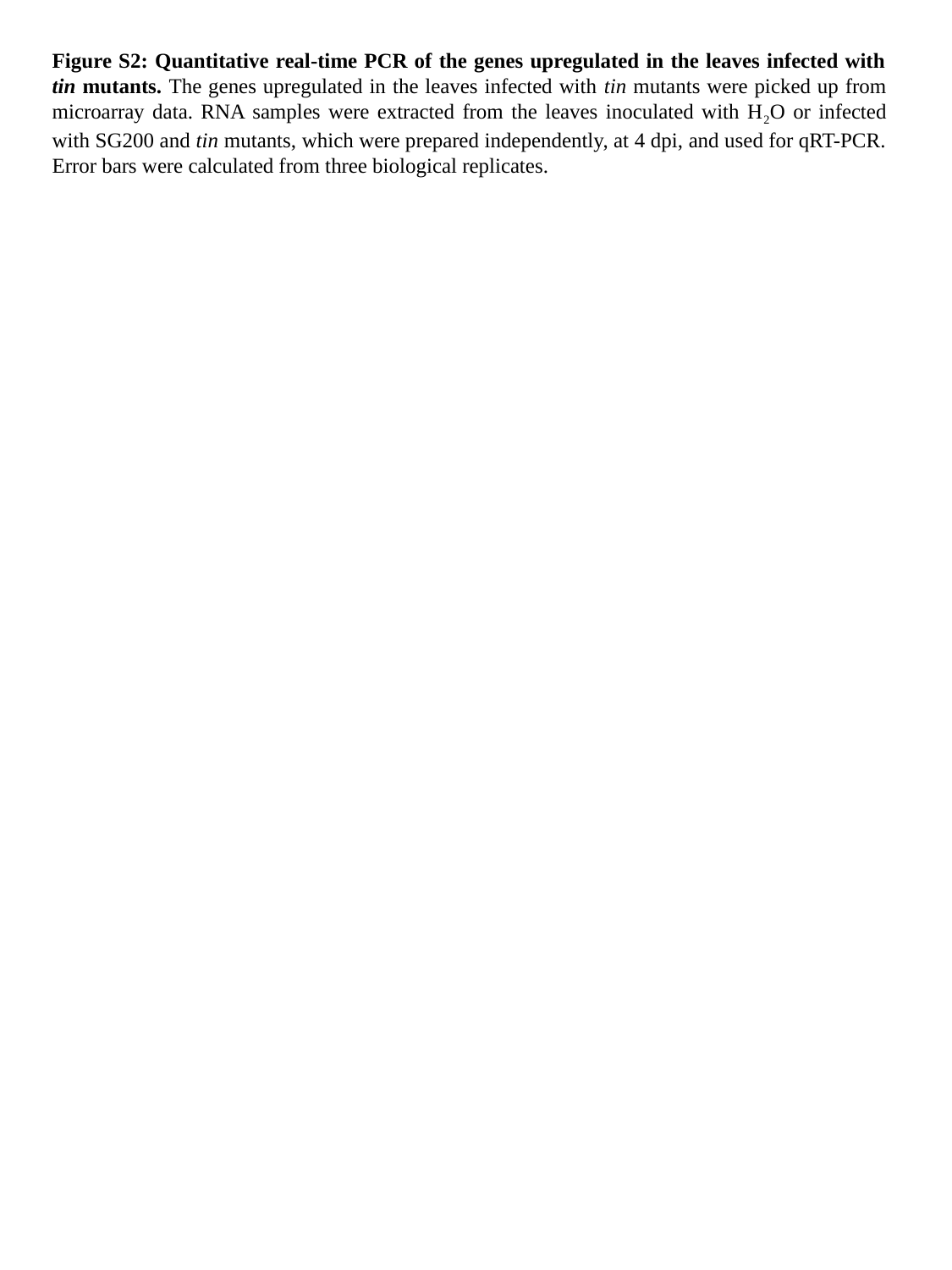

Figure S2: Quantitative real-time PCR of the genes upregulated in the leaves infected with tin mutants. The genes upregulated in the leaves infected with tin mutants were picked up from microarray data. RNA samples were extracted from the leaves inoculated with H2O or infected with SG200 and tin mutants, which were prepared independently, at 4 dpi, and used for qRT-PCR. Error bars were calculated from three biological replicates.
