# Supplementary material for: Characterization of the Largest Effector Gene Cluster of Ustilago maydis
Source: PLoS Pathog. 2014 Jul 3;10(7):e1003866. doi: 10.1371/journal.ppat.1003866 (PMC4081774; doi:10.1371/journal.ppat.1003866)
Supplement: Figure S3 — Gene ontology enrichment analysis of maize genes induced by the cluster 19A deletion but not by the tin mutants at 4 dpi. The GOEAST software toolkit [43] was used to identify GO terms for cellular processes (yellow boxes) that are specifically enriched in maize leaves infected with U. maydis strain SG200Δ19A samples. Darker color shades indicate higher significance of enrichment. p-values are indicated in brackets. (PPTX) [file ppat.1003866.s003.pptx]

## Slide 1
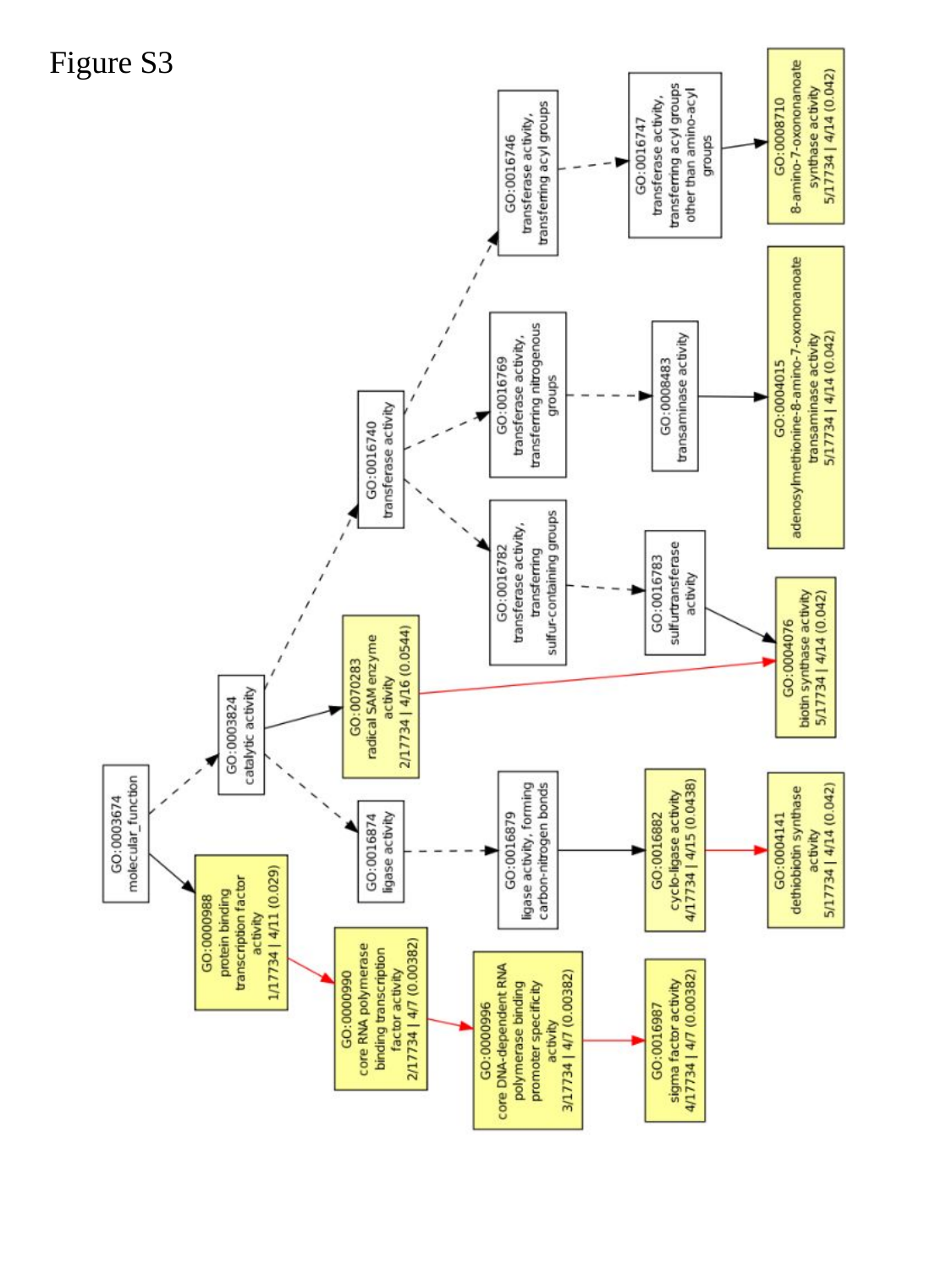

Figure S3

## Slide 2
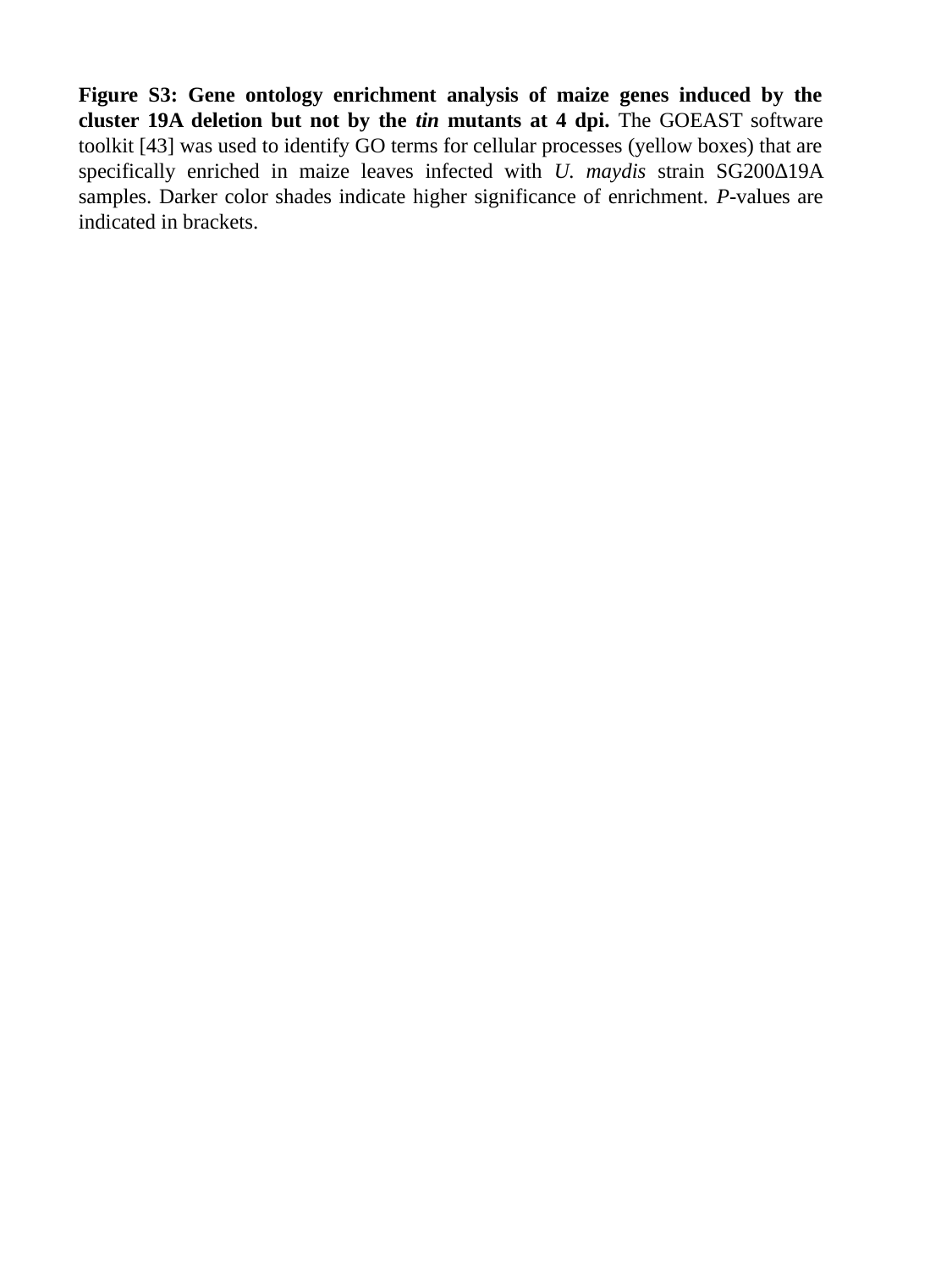

Figure S3: Gene ontology enrichment analysis of maize genes induced by the cluster 19A deletion but not by the tin mutants at 4 dpi. The GOEAST software toolkit [43] was used to identify GO terms for cellular processes (yellow boxes) that are specifically enriched in maize leaves infected with U. maydis strain SG200∆19A samples. Darker color shades indicate higher significance of enrichment. P-values are indicated in brackets.
