# Supplementary material for: Characterization of the Largest Effector Gene Cluster of Ustilago maydis
Source: PLoS Pathog. 2014 Jul 3;10(7):e1003866. doi: 10.1371/journal.ppat.1003866 (PMC4081774; doi:10.1371/journal.ppat.1003866)
Supplement: Figure S5 — Gene ontology enrichment analysis of maize genes downregulated by the tin mutants and the cluster 19A deletion at 4 dpi. The GOEAST software toolkit [43] was used to identify GO terms for cellular processes that are enriched in maize leaves infected with U. maydis strain SG200Δ19A and the tin mutants compared to mock-treated samples. Yellow boxes indicate processes enriched in both SG200Δ19A and the tin mutants infected samples. Darker color shades indicate higher significance of enrichment. p-values are indicated in brackets. (PPTX) [file ppat.1003866.s005.pptx]

## Slide 1
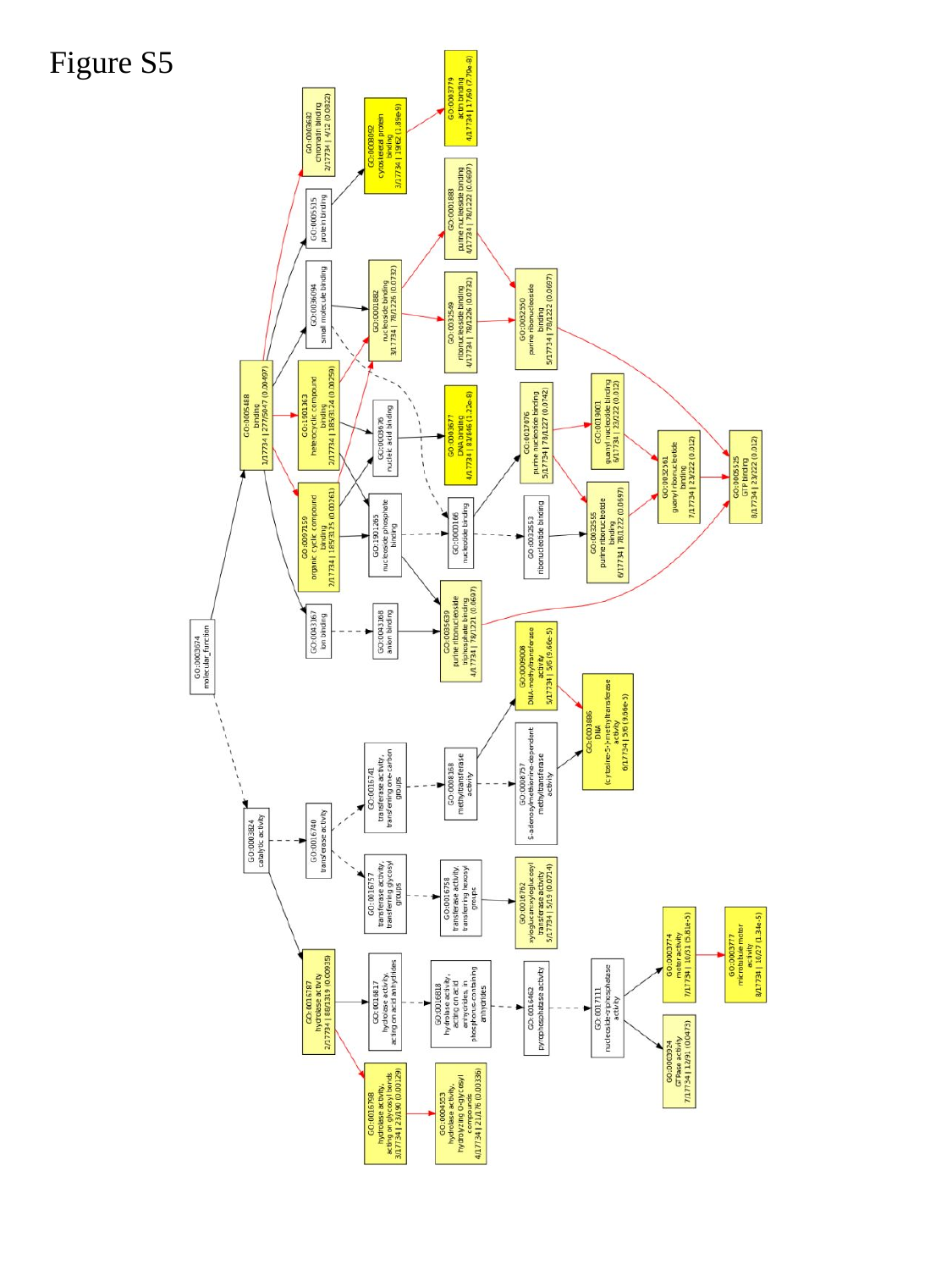

Figure S5

## Slide 2
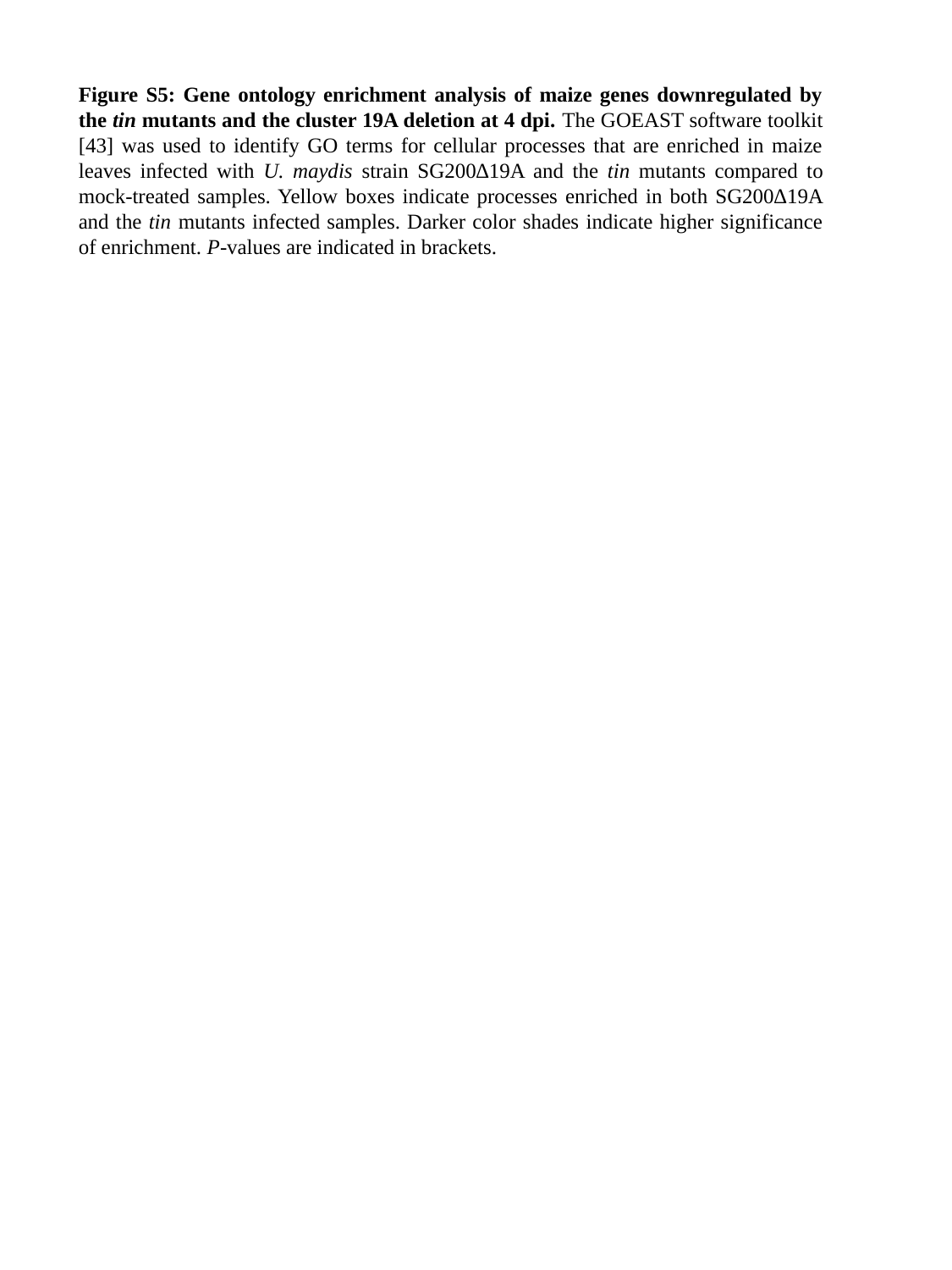

Figure S5: Gene ontology enrichment analysis of maize genes downregulated by the tin mutants and the cluster 19A deletion at 4 dpi. The GOEAST software toolkit [43] was used to identify GO terms for cellular processes that are enriched in maize leaves infected with U. maydis strain SG200∆19A and the tin mutants compared to mock-treated samples. Yellow boxes indicate processes enriched in both SG200∆19A and the tin mutants infected samples. Darker color shades indicate higher significance of enrichment. P-values are indicated in brackets.
