# Supplementary material for: Characterization of the Largest Effector Gene Cluster of Ustilago maydis
Source: PLoS Pathog. 2014 Jul 3;10(7):e1003866. doi: 10.1371/journal.ppat.1003866 (PMC4081774; doi:10.1371/journal.ppat.1003866)
Supplement: Figure S6 — Gene ontology enrichment analysis of maize genes induced after infection with U. maydis strain SG200Δtin3 at 4 dpi. The GOEAST software toolkit [43] was used to identify GO terms for cellular processes (yellow boxes) that are specifically enriched in maize leaves infected with U. maydis strain SG200Δtin3 Darker color shades indicate higher significance of enrichment. p-values are indicated in brackets. (PPTX) [file ppat.1003866.s006.pptx]

## Slide 1
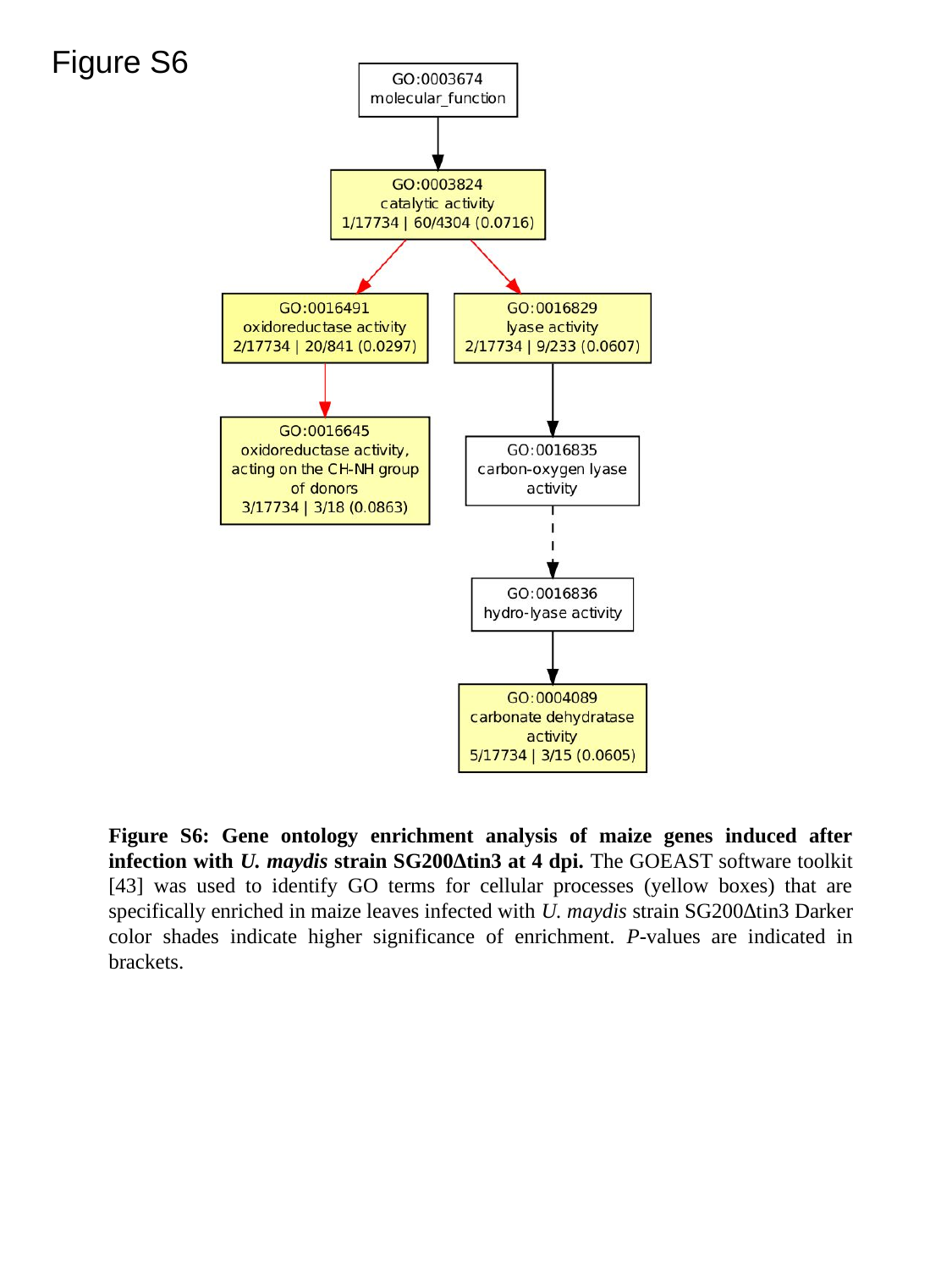

Figure S6
Figure S6: Gene ontology enrichment analysis of maize genes induced after infection with U. maydis strain SG200∆tin3 at 4 dpi. The GOEAST software toolkit [43] was used to identify GO terms for cellular processes (yellow boxes) that are specifically enriched in maize leaves infected with U. maydis strain SG200∆tin3 Darker color shades indicate higher significance of enrichment. P-values are indicated in brackets.
