# Supplementary material for: Characterization of the Largest Effector Gene Cluster of Ustilago maydis
Source: PLoS Pathog. 2014 Jul 3;10(7):e1003866. doi: 10.1371/journal.ppat.1003866 (PMC4081774; doi:10.1371/journal.ppat.1003866)
Supplement: Figure S8 — Gene ontology enrichment analysis of maize genes induced after infection with U. maydis strain SG200Δtin5 at 4 dpi. The GOEAST software toolkit [43] was used to identify GO terms for cellular processes (yellow boxes) that are specifically enriched in maize leaves infected with U. maydis strain SG200Δtin5. Darker color shades indicate higher significance of enrichment. p-values are indicated in brackets. (PPTX) [file ppat.1003866.s008.pptx]

## Slide 1
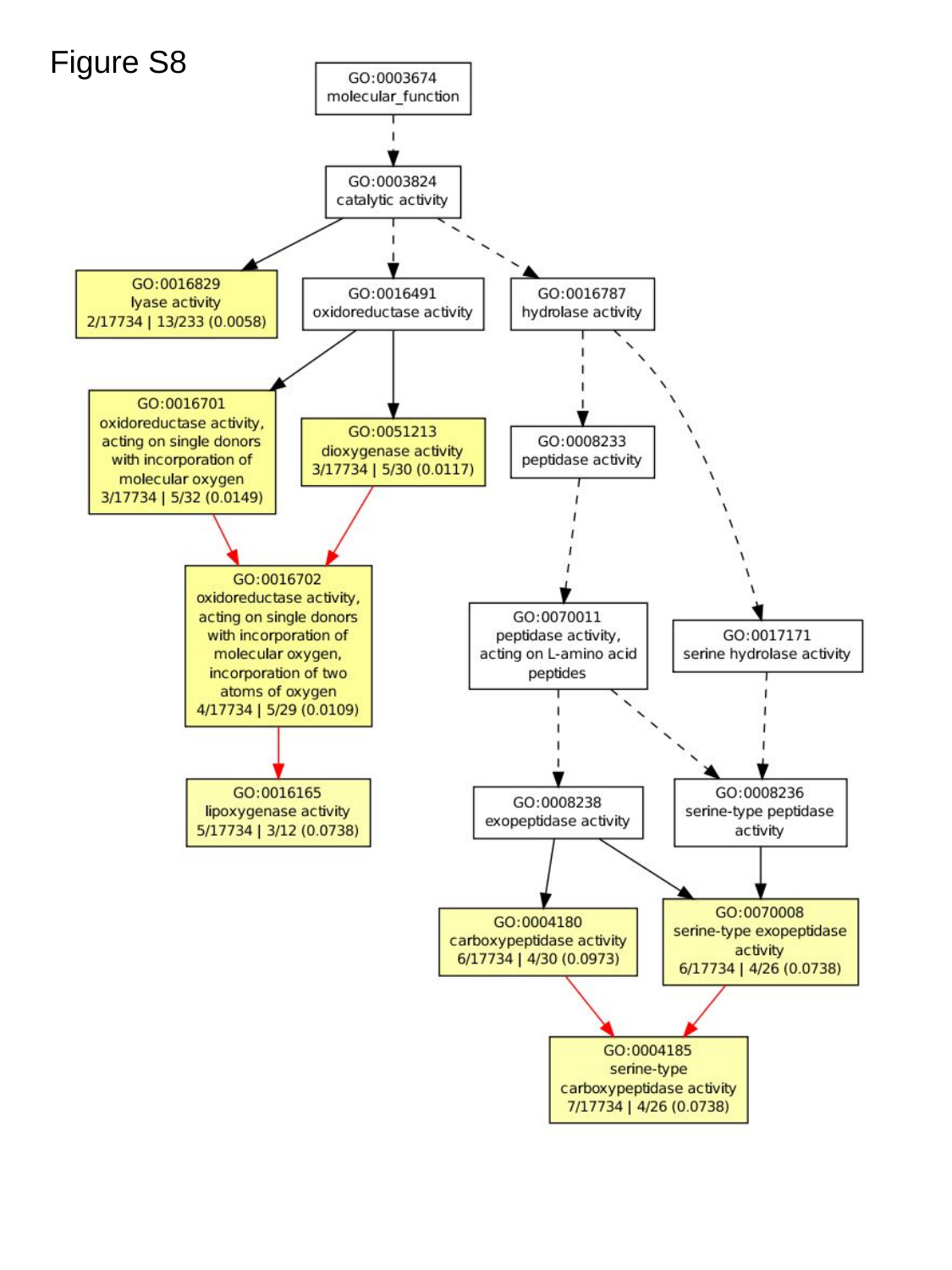

Figure S8

## Slide 2
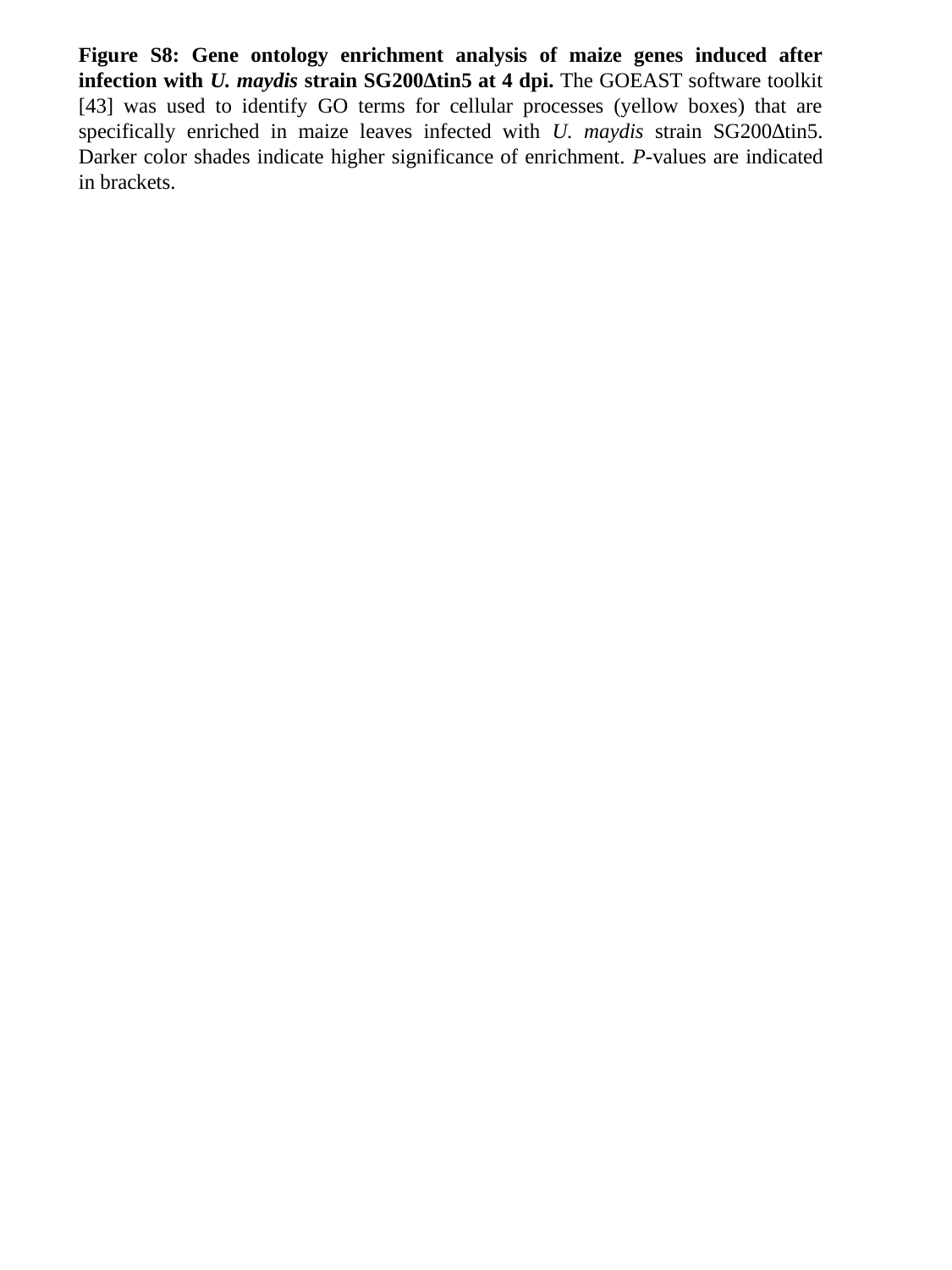

Figure S8: Gene ontology enrichment analysis of maize genes induced after infection with U. maydis strain SG200∆tin5 at 4 dpi. The GOEAST software toolkit [43] was used to identify GO terms for cellular processes (yellow boxes) that are specifically enriched in maize leaves infected with U. maydis strain SG200∆tin5. Darker color shades indicate higher significance of enrichment. P-values are indicated in brackets.
