# Supplementary material for: Characterization of the Largest Effector Gene Cluster of Ustilago maydis
Source: PLoS Pathog. 2014 Jul 3;10(7):e1003866. doi: 10.1371/journal.ppat.1003866 (PMC4081774; doi:10.1371/journal.ppat.1003866)
Supplement: Table S5 — U. maydis strains used in this study. (DOCX) [file ppat.1003866.s013.docx]

Supplementary Table 5 *U. maydis* strains used in this study

| **Strain^1^** | **Genotype** | **Resistance^2^** | **Reference** |
| --- | --- | --- | --- |
| SG200 | *a1:mfa2 bW2bE1* | P | [[1](#_ENREF_1)2] |
| SG200Δ19A | *a1:mfa2 bW2bE1 Δum05294-um05319* | P, H | [[1](#_ENREF_1)2] |
| SG200Δ19A-1 | *a1:mfa2 bW2bE1 Δum05294-um10556* | P, N | this study |
| SG200Δ19A-2 | *a1:mfa2 bW2bE1 Δum05308-um05319* | P, H | this study |
| SG200Δ19A-1a | *a1:mfa2 bW2bE1 Δum05299-um05301* | P, H | this study |
| SG200Δ19A-1b | *a1:mfa2 bW2bE1 Δum05294-um10554* | P, N | this study |
| SG200Δ19A-1c | *a1:mfa2 bW2bE1 Δum05302-um10555* | P, N | this study |
| SG200Δ19A-1d | *a1:mfa2 bW2bE1 Δum05305-um10556* | P, N | this study |
| SG200Δ19A-2e | *a1:mfa2 bW2bE1 Δum05318-um05319* | P, H | this study |
| SG200Δ19A-1bcd | *a1:mfa2 bW2bE1 Δum05294-um10554 Δum05302-um10555 Δum05305-um10556* | P, H, N, C | this study |
| SG200Δ*tin2* | *a1:mfa2 bW2bE1 Δum05302* | P, N | this study |
| SG200Δ*tin3* | *a1:mfa2 bW2bE1 Δum10556* | P, H | this study |
| SG200Δ*tin4* | *a1:mfa2 bW2bE1 Δum05318* | P, H | this study |
| SG200Δ*tin5* | *a1:mfa2 bW2bE1 Δum05319* | P, H | this study |
| SG200Δ*tin2-tin2* | *a1:mfa2 bW2bE1 Δum05302p^R^[um05302]ip^S^* | P, N, C | this study |
| SG200Δ*tin3-tin3* | *a1:mfa2 bW2bE1 Δum10556p^R^[um10556]ip^S^* | P, H, C | this study |
| SG200Δ*tin4-tin4** | *a1:mfa2 bW2bE1 Δum05318p^R^[um05318]ip^S^* | P, H, C | this study |
| SG200Δ*tin5-tin5** | *a1:mfa2 bW2bE1 Δum05319p^R^[um05319]ip^S^* | P, H, C | this study |
| SG200Δ19A-1b-19A-1b* | *a1:mfa2 bW2bE1 Δum05294-um10554 p^R^[um05294-um10554]ip^S^* | P, H, C | this study |

^1^ Double integrations are marked with *.

^2^ Phleomycin (P), Hygromycin (H), Nourseothricin (N), Carboxin (C).
