# Supplementary material for: Characterization of the Largest Effector Gene Cluster of Ustilago maydis
Source: PLoS Pathog. 2014 Jul 3;10(7):e1003866. doi: 10.1371/journal.ppat.1003866 (PMC4081774; doi:10.1371/journal.ppat.1003866)
Supplement: Table S6 — PCR primers used in this study. (DOCX) [file ppat.1003866.s014.docx]

Supplementary Table 6

PCR primers used in this study

| **Name** | **Sequence 5’ -> 3’^1^** | **Application^2^** |
| --- | --- | --- |
| 5294-L-uni | TCTGACAGCAACGGTCTGTTTAGG | Deletion construct of cluster 19A, 19A-1, 19A-1b [F] |
| 5294-ORF-linker-SfiF-rev | cacggccgcgttggccccggtggcgatcgagcgaaggaacaaagcggctcgtagcatc | Deletion construct of cluster 19A, 19A-1, 19A-1b [R] |
| 5319-R-rev | ATTCACCGTTGATGTAGTTGTCGG | Deletion construct of cluster 19A, 19A-2, 19A-2e [R] |
| 5319-R-SfiI-uni | cacggcctgagtggcccgagagatatgaccatatccttcg | Deletion construct of cluster 19A, 19A-2, 19A-2e [F] |
| 10556-R-rev | cctgttgacaaatgaaggtcaagc | Deletion construct of cluster 19A-1, 19A-1d and of *tin3* [R] |
| 10556-R-SfiI-uni | cacggcctgagtggccggaaagagtcttgagatgtgcaagc | Deletion construct of cluster 19A-1, 19A-1d and of *tin3* [F] |
| 5308-L-uni | gcctatgatggctttaagcgttcg | Deletion construct of cluster 19A-2 [F] |
| 5308-L-SfiI-rev | gtgggccgcgttggccattcaacgtgcgtggagaatgctg | Deletion construct of cluster 19A-2 [R] |
| 5299-L-uni | atcgtcgctaggctagccatcgcc | Deletion construct of cluster 19A-1a [F] |
| 5299-L-SfiI-rev | gtgggccgcgttggccggttcaaagctaccctttcgaggacg | Deletion construct of cluster 19A-1a [R] |
| 5301-R-rev | actgtgatgccgttggcgagaactc | Deletion construct of cluster 19A-1a [R] |
| 5301-R-SfiI-uni | cacggcctgagtggccctatgggcgcacgatgctacctctgc | Deletion construct of cluster 19A-1a [F] |
| 10554-R-rev | gctgctgttgttagaacgaagagc | Deletion construct of cluster 19A-1b [R] |
| 10554-R-SfiI-uni | cacggcctgagtggccggctctcggttgcgctgtgtgc | Deletion construct of cluster 19A-1b [F] |
| 5302-L-uni2 | ccaggagataaggttgttcatcc | Deletion construct of cluster 19A-1c and of *tin2* [F] |
| 5302-L-SfiI-rev | gtgggccatctaggccggagctttgaatgggagaatgagg | Deletion construct of cluster 19A-1c and of *tin2* [R] |
| 10555-R-rev | catcactcgctgatgattcagcg | Deletion construct of cluster 19A-1c [R] |
| 10555-R-SfiI-uni | cacggcctgagtggccgcacgaaatggtttttcaggtagc | Deletion construct of cluster 19A-1c [F] |
| 5305-L-uni | ggtccattgcaacaacacgtaagc | Deletion construct of cluster 19A-1d [F] |
| 5305-L-SfiI-rev | gtgggccatctaggcctcaagctcgaggaggcgac | Deletion construct of cluster 19A-1d [R] |
| 5318-L-uni | Gctcgagcatcttagtccgttagc | Deletion construct of cluster 19A-2e [F] |
| 5318-L-SfiI-rev | Gtgggccatctaggccgagtgaagctaacggactaagatgc | Deletion construct of cluster 19A-2e [R] |
| 5302-R-rev | Gacagttgtcggaattggtcaagg | Deletion construct of *tin2* [R] |
| 5302-R-SfiI-uni | cacggcctgagtggccaagcctcacagacagtatagatgc | Deletion construct of *tin2* [F] |
| 5306-ORF-uni | gcaacacctgacacagcgcagtg | Deletion construct of *tin3* [F] |
| 10556-L-SfiI-rev | gtgggccatctaggccactcactcgaagtcgtgtaacc | Deletion construct of *tin3* [R] |
| 5318-R-rev | ccgtgattccaaactgtctctgc | Deletion construct of *tin4* [R] |
| 5318-R-SfiI-uni | cacggcctgagtggccgcacttctagtatcagcaccaattgc | Deletion construct of *tin4* [F] |
| 5319-L-uni | Gctcgagcatcttagtccgttagc | Deletion construct of *tin5* [F] |
| 5319-L-SfiI-rev | Gtgggccatctaggccttggaaggtatcagagtaagcttgc | Deletion construct of *tin5* [R] |
| 5302-Prom-SpeI-uni | cgactagtctatgggcgcacgatgctacc | Complementation construct of *tin2* with native promoter [F] |
| 5302_ORF_AscI_rev | Ttggcgcgcctcaaagagggaagcgagggagc | Complementation construct of *tin2* with native promoter [R] |
| 10556-Prom-XbaI-uni | ttgtggcaagactctagatacg | Complementation construct of *tin3* with native promoter [F] |
| 10556-ORF-AscI-rev | Ttggcgcgccttacgatgacgttccctcatcg | Complementation construct of *tin3* with native promoter [R] |
| 5318-Prom-SpeI-uni | Attgcgactagtctatctcacacgccgaattgatgg | Complementation construct of *tin4* with native promoter [F] |
| 5318-ORF-AscI-rev | Attggcgcgcctagaagtgcaaagcaagtgagcc | Complementation construct of *tin4* with native promoter [R] |
| 5319-Prom-XbaI-uni | Taatctagacacttctagtatcagcaccaattgc | Complementation construct of *tin5* with native promoter [F] |
| 5319-ORF-AscI-rev | Attggcgcgcctagatttctggcacctcgggg | Complementation construct of *tin5* with native promoter [R] |
| 5294-Prom-SpeI-uni | gcgactagtctgctcaaacgcattcggacaacg | Complementation construct of 19A-1b with native promoter of *um05294* [F] |
| 10554-ORF-AscI-rev | Attggcgcgccctaaacaggatgatgttcaagaacc | Complementation construct of 19A-1b with native promoter of *um05294* [R] |
| GAPDH-F | CTTCGGCATTGTTGAGGGTTTG | Amplification of *gapdh* of *Z. mays* for qPCR [F] |
| GAPDH-R | TCCTTGGCTGAGGGTCCGTC | Amplification of *gapdh* of *Z. mays* for qPCR [R] |
| PPI-fw | ACATCGTCAAGGCTATCG | Amplification of *ppi* of *U. maydis* for qPCR [F] |
| PPI-re | AAAGAACACCGGACTTGG | Amplification of *ppi* of *U. maydis* for qPCR [R] |
| RT-Endo_fw | ATGGACGAGGACCCATACAG | Amplification of NM_001154745 for qRT-PCR [F] |
| RT-Endo_re | AGAACCAGATGGCCGTCTTG | Amplification of NM_001154745 for qRT-PCR [R] |
| RT-prp4_fw | ACTACGTGGACCCGCACAAC | Amplification of NM_001111929 for qRT-PCR [F] |
| RT-prp4_re | GTGTCGTGGTCGTAGTACTG | Amplification of NM_001111929 for qRT-PCR [R] |
| RT-beta_fw | CCGCCATGCAGAACCTCAAC | Amplification of Loc100280140 for qRT-PCR [F] |
| RT-beta_re | CCATGTAGCCCTGCGAGAAG | Amplification of Loc100280140 for qRT-PCR [R] |
| RT-kinase_fw | GGTACGCGCTCATCCTCATC | Amplification of NM_001154030 for qRT-PCR [F] |
| RT-kinase_re | TCGGAAGCGGCAGTAGTTGG | Amplification of NM_001154030 for qRT-PCR [R] |
| RT-Sucro_fw | GAAACCGACAAGAGACTCAC | Amplification of NM_001111941 for qRT-PCR [F] |
| RT-Sucro_re | AGGCCTGTCATGTTCTTCAC | Amplification of NM_001111941 for qRT-PCR [R] |
| RT-Terp_fw | GGAACCGAACAAGAAGTACC | Amplification of NM_001154674 for qRT-PCR [F] |
| RT-Terp_re | CCGAAGCCCATTAGTGTTAG | Amplification of NM_001154674 for qRT-PCR [R] |
| RT-GT6_fw | GCTCAAGTCCACGTTCAAGG | Amplification of NM_001111694 for qRT-PCR [F] |
| RT-GT6_re | AAGTAGTAGCCGCTCTCCAG | Amplification of NM_001111694 for qRT-PCR [R] |
| RT-Perox_fw | TGACGGTGCTGGAGCAGAAG | Amplification of NM_001143372 for qRT-PCR [F] |
| RT-Perox_re | GGTTCGTGCCGCTGAAGTTG | Amplification of NM_001143372 for qRT-PCR [R] |
| RT-Inver_fw | AGCCGGGACTTCAGGAAGTG | Amplification of NM_001111899 for qRT-PCR [F] |
| RT-Inver_re | TGAGGTCGAGGCTGCTCTTG | Amplification of NM_001111899 for qRT-PCR [R] |
| RT-Lipo_fw | TGTCGCCGTCAACGACTCTG | Amplification of NM_001112045 for qRT-PCR [F] |
| RT-Lipo_re | TGGTCATGGTGTCGCGGAAG | Amplification of NM_001112045 for qRT-PCR [R] |
| RT-bras_fw | ATCCCAACTACGCTTGGTTC | Amplification of NM_001153886 for qRT-PCR [F] |
| RT-bras_re | TCAGTTCCACAAGGCTAGTC | Amplification of NM_001153886 for qRT-PCR [R] |
| RT-TF_fw | AGGAGATGCCGCATAAGAGC | Amplification of NM_001153429 for qRT-PCR [F] |
| RT-TF_fw | ACTTGTCGCACCTCCTGTTC | Amplification of NM_001153429 for qRT-PCR [R] |
| RT-cyto_fw | CTATACTGCCTCGAGCTAAC | Amplification of NM_001112056 for qRT-PCR [F] |
| RT-cyto_re | CGGTGTGGAATAGAGTAGAC | Amplification of NM_001112056 for qRT-PCR [R] |

^1^ Sequences are shown in 5‘ to 3‘ direction; Restriction sites are underlined.

^2^ Oligonucleotides hybridize with the sense-strand [R] or with the complementary strand [F] of the corresponding gene.
