# Supplementary material for: Characterization of the Largest Effector Gene Cluster of Ustilago maydis
Source: PLoS Pathog. 2014 Jul 3;10(7):e1003866. doi: 10.1371/journal.ppat.1003866 (PMC4081774; doi:10.1371/journal.ppat.1003866)
Supplement: Table S7 — Plasmids used in this study. (DOCX) [file ppat.1003866.s015.docx]

Supplementary Table 7

Plasmids used in this study

| **Plasmid** | **Reference** |
| --- | --- |
| pCRII_D19A-3_hyg | This study |
| pCR2.1_D19A-1_nat | This study |
| pCRII_D19A-2_hyg | This study |
| pCR2.1_D19A-1a_hyg | This study |
| pCR2.1_D19A-1b_nat | This study |
| pCR2.1_D19A_1b_hyg | This study |
| pCRII_D19A-1c_nat | This study |
| pCRII_D19A-1d_nat | This study |
| pCRII_D19A-1d_cbx | This study |
| pCRII_D19A-2e_hyg | This study |
| pCR2.1_D*tin2*_nat | This study |
| pCRII_D*tin3*_hyg | This study |
| pCRII_D*tin4*_hyg | This study |
| pCRII_D*tin5*_hyg | This study |
| pIP:P*um05294*-*um10554* | This study |
| pIP:P*um05302* | This study |
| pIP:P*um10556* | This study |
| pIP:P*um05318* | This study |
| pIP:P*um05319* | This study |
